# Systematic identification of *Salmonella* T6SS effectors uncovers diverse new families and lipid-targeting activities

**DOI:** 10.1371/journal.pbio.3003680

**Published:** 2026-03-17

**Authors:** Gianlucca G. Nicastro, Stephanie Sibinelli-Sousa, Julia T. Hespanhol, Thomas W. C. Santos, Joseph P. Munoz, Rosangela S. Santos, Blanca M. Perez-Sepulveda, Sayuri Miyamoto, L. Aravind, Robson F. de Souza, Ethel Bayer-Santos

**Affiliations:** 1 Departamento de Microbiologia, Instituto de Ciências Biomédicas, Universidade de São Paulo, São Paulo, Brazil; 2 Computational Biology Branch, National Center for Biotechnology Information, National Library of Medicine, National Institutes of Health, Bethesda, Maryland, United States of America; 3 Department of Molecular Biosciences, College of Natural Sciences, The University of Texas at Austin, Austin, Texas, United States of America; 4 Departamento de Bioquímica, Instituto de Química, Universidade de São Paulo, São Paulo, Brazil; 5 Institute of Infection, Veterinary and Ecological Sciences, University of Liverpool, Liverpool, United Kingdom; 6 LaMontagne Center for Infectious Disease, The University of Texas at Austin, Austin, Texas, United States of America; Massachusetts Institute of Technology, Howard Hughes Medical Institute, UNITED STATES OF AMERICA

## Abstract

Bacterial warfare is a widespread phenomenon in which bacteria deploy toxins to inhibit or kill competitors. These toxins disrupt essential cellular processes, and their diversification is driven by an evolutionary arms race involving toxin and immunity gene acquisition. Here, we used *in-silico* approaches to analyze genomes from the 10k *Salmonella* Project and identify effectors secreted via the Type VI Secretion System (T6SS). We uncovered 128 candidates distributed across diverse *Salmonella* serovars and other bacterial species. Among them, Tox-Act1 was selected for in-depth characterization. Tox-Act1 contains a permuted NlpC/P60 papain-like catalytic core typical of lipid-targeting enzymes. Evolutionary analysis revealed its relationship with acyltransferases. Biochemical assays and lipidomics of intoxicated cells showed that Tox-Act1 acts as a phospholipase, cleaving phosphatidylglycerol and phosphatidylethanolamine. We further demonstrate that Tox-Act1 is secreted in a T6SS-dependent manner and provides a competitive advantage during mouse gut colonization. This study broadens our understanding of toxin domain diversity and provides the first direct characterization of a lipid-targeting NlpC/P60 toxin domain.

## Abstract 2

During microbial warfare, bacteria deploy toxins to inhibit or kill competitors. This study reveals lipid-targeting antibacterial toxins and previously unknown toxin domains within Salmonella T6SS effectors, broadening our understanding of enzymatic diversity and the molecular strategies bacteria use to compete in the gut.

## Introduction

Competition is a fundamental biological process in nature, occurring both within and between species that share a common environment. Bacteria actively participate in these ecological battles employing a potent arsenal of toxins as their weaponry [1]. A prominent mechanism for toxin delivery among gram-negative bacteria is via the Type VI secretion system (T6SS) [2]. Phylogenetic analysis of T6SS components showed that there are four types of T6SSs (T6SS^i–iv^) [3–5], with the canonical T6SS^i^ present in *Proteobacteria* being further classified into six subtypes (i1, i2, i3, i4a, i4b, and i5) [4,6,7]. The T6SS functions in a contact-dependent manner and relies on the biochemical properties of secreted effector toxins for its function [8]. These toxin domains frequently recombine and move via lateral gene transfer, allowing them to be delivered by different secretion systems, which warrant their name as polymorphic toxins [9].

During secretion via the T6SS, toxins are loaded onto a spear-like structure formed by hexameric rings of Hcp proteins capped by a spike comprising a trimer of VgrG sharpened by a PAAR protein [10]. Effectors are translocated into target cells either fused at the C-terminus of Hcp, VgrG, and PAAR proteins (named specialized effectors), or associated with these proteins via adaptors (cargo effectors) [11]. Antibacterial toxins are often paired with immunity proteins that prevent self-intoxication, thus forming gene pairs that are frequently located near structural components of the T6SS [8,12].

The protective role of the endogenous microbiota against *Salmonella* infection has been recognized for years [13]; however, only recently studies have started to reveal the mechanism by which the microbiota maintains gut homeostasis and promotes colonization resistance [14]. Despite this progress, there is still limited understanding of the direct antimicrobial strategies employed by commensals and pathogens during these disputes for niche control [15]. T6SSs clusters are conserved across several *Salmonella* spp., highlighting their importance for bacterial fitness [16,17]. These systems are encoded in distinct pathogenicity islands (SPIs), acquired through independent horizontal gene transfer events [16,17]. *S.* Typhimurium, for example, encodes a T6SS subtype i3 within SPI-6, which is important for interbacterial competition and gut colonization in mice [18,19]. While some studies have begun to catalog candidate T6SS effectors in *Salmonella* [20–22], the identity and mechanisms of action of most effectors remain poorly defined.

Here, we set out to identify the repertoire of T6SS effectors in a dataset of isolates from the 10k *Salmonella* Genomes project using a computational approach. Employing sensitive sequence and structure searches alongside comparative genomics, we identified 128 candidates that are widespread among several *Salmonella* serovars and additional bacterial species. This comprehensive analysis indicates that T6SSs subtypes i3 and i1 are associated with antibacterial and anti-eukaryotic effectors, respectively. Furthermore, our findings reveal that each of the 149 serovars harbors a unique combination of effectors, likely required for their specific ecological interactions. A detailed examination of a selected candidate (Tox-Act1) encoding a newly identified domain showed that it is an antibacterial effector belonging to the NlpC/P60 superfamily with a permuted catalytic core. Tox-Act1 is evolutionarily related to acyltransferases and displays phospholipase activity to modify target-cell membrane composition. This study offers a comprehensive characterization of new toxins, especially the arsenal linked to T6SSs in *Salmonella*, identifying novel toxin domains and providing an in-depth analysis of a new protein family that specifically targets lipids.

## Results

### Computational pipeline for the identification of T6SS components

**Fig 1:**
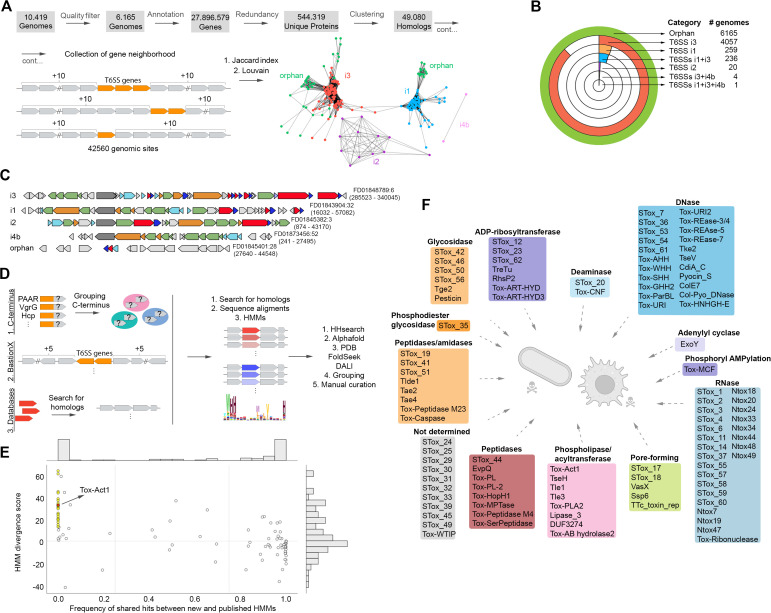
Computational pipeline for the identification of T6SS subtypes and effector repertoire within 10,000 *Salmonella* genomes. **(a)** Pipeline used for classification of genomic sites and T6SS subtypes. **(b)** Number of genomes containing the different T6SS subtypes within the 10KSG dataset. **(c)** Examples of the genomic organization of T6SS structural clusters from distinct phylogenetic subtypes. Colors denote structural proteins forming the membrane complex (orange), sheath and inner tube (light blue), baseplate and spike components (green), *clpV* is in dark gray. Effectors are shown in red and immunity proteins in dark blue. Accessory or genes encoding proteins unrelated to T6SS are in light gray. **(d)** *In silico* strategies used for the identification and classification of T6SS effectors. **(e)** Comparison between *Salmonella* effector profile Hidden Markov Models (pHMMs) and previously published models of related protein families. Each circle represents a group of protein hits identified using an STox model as query against the NR50 database with HMMER 3.3. The x-axis indicates the relative frequency of hits shared between the STox model and its closest-matching reference model. Yellow circles highlight STox models that detect proteins not captured by any existing models. As the STox hits diverge from the proteins represented by the reference model, the e-values of the alignments of reference models to STox hits will increase, resulting in higher HMM divergence. STox models representing novel protein families tend to cluster on the left side of the plot (shared hit frequency ≤25%). **(f)** Schematic representation of the functional classes of T6SS effectors identified in the 10KSG. Created with BioRender.com. The data and code needed to generate this figure can be found in https://doi.org/10.5281/zenodo.18590644.

T6SS effectors are often encoded in close genomic proximity to structural components of the system [16,23,24]. To identify new effectors, we employed a “guilt-by-association” approach, which relies on the conservation of genomic context [25]. We applied this methodology to 10,419 genomes of *Salmonella* isolates sequenced by the 10KSG Consortium [26] (Fig 1A). Using gene annotations provided by the 10KSG (https://doi.org/10.5281/zenodo.18590644), we extracted and clustered the full proteome of these isolates, generating a total of 49,080 sequence similarity groups. We then identified all proteins with significant similarity to T6SS component genes using hidden Markov models (HMMs) from different sources [9,27–29]. We collected 10 genes upstream and downstream of each locus encoding putative T6SS components, referred to as genomic sites (Fig 1A). A total of 42,560 genomic sites housing at least one T6SS component were identified. We then used the sequence similarity groups of the proteins as labels for genes in genomic sites, thus representing each genomic site as a set of labels. Based on this representation, the Jaccard distance was used to measure the similarity between pairs of genomic sites and a network of loci using this distance as edge weights was built [30]. By applying the community detection Louvain algorithm [31], we generated 46 unsupervised groups of genomic sites. Analysis of gene composition at genomic sites (S1 Table) and comparison of the phylogenies of several T6SS components (S1 Data) resulted in the assignment of genomic sites to four T6SS subtypes (i1, i2, i3, i4b), an orphan category and the identification of tailocin/phages loci (Fig 1B and 1C; S1 Table and S1 Data). Once identified, tailocins/phages were removed from our dataset and not considered for further analysis.

We observed mobility of the distinct T6SS subtypes among genomic sites and pathogenicity islands, depending on the strain and/or serovar. Using tRNAs as markers to identify the insertion sites [32], we found that subtype i3 is predominantly located within SPI-6, flanked by the aspartate tRNA (tRNA^Asp^), accounting for 90% of cases (4,929 out of 5,461 genomic sites) [16]. In contrast, subtype i1 is more variable: it is found within SPI-19 associated with tRNA^Ala^ in 12% of cases (66 out of 532 genomic sites), and within SPI-6 flanked by tRNA^Asp^ in 3% of cases (18 out of 532 genomes). However, due to the high fragmentation of most of the genome assemblies analyzed, the majority of subtype i1 instances (82%, 437 out of 532 genomic sites) could not be assigned to a specific genomic location. These results indicate that distinct T6SSs subtypes are inserted into different genomic sites that are hot spots for horizontal transfer events, and the combination between the insertion site and the introduced subtype varies according to the *Salmonella* strain/serovar.

### Identification of the T6SS effectors repertoire in the 10KSG dataset

Next, we focused on identifying effector toxins using three strategies (Fig 1D). First, we focused on proteins containing N-terminal PAAR, VgrG, or Hcp domains and additional C-terminal domains with more than 50 amino acids. These C-terminal regions were isolated and grouped based on similarity (80% coverage and 1e−3 e-value). Second, we used the genomic sites containing T6SS components and analyzed up to five genes upstream and downstream via the software BastionX [33]. Third, we used amino acid sequences, HMMs, and PSSMs (position-specific scoring matrix) from SecReT6 [34], Pfam [28], and Zhang and colleagues [9] to search the 10KSG dataset for previously described effectors. For candidates not recognized by previously described models, we collected homologs from NCBI using 4 iterations of PSI–BLAST [35] and generated multiple sequence alignments and HMMs. To distinguish putative new toxin domains from false positives, we collected homologs across multiple taxa and analyzed their genomic context. We then followed the principle underling the classification of polymorphic toxin domains [9]: if a homolog consistently appears fused to secretion-related domains or within secretion system genomic loci, and it is consistently accompanied by a putative cognate protein that has characteristics of immunity proteins, this new domain is highly likely to function in interbacterial antagonism.

We then used a series of sequence and structure-based strategies to classify and annotate the function of these candidates: (i) profile-profile comparison methods such as HHsearch [36] were used to detect distant homologs; (ii) structural models were created using Alphafold2 [37] from multiple sequence alignments to perform searches in FoldSeek [38] and DALI [39] against PDB [40] and AlphaFoldDB [41]; (iii) the structure-structure comparison algorithm from FoldSeek was used to cluster groups of candidates. All information collected was manually examined to establish the final domain annotation (Fig 1D). Candidates that displayed sequence or structural similarity to known toxin domains or proteins of unknown function that displayed conserved genomic organization and/or adjacent conserved putative immunity proteins across several species were maintained. We note that our approach was not exhaustive and some effectors that are consistently unlinked from a T6SS loci across the 10KSG may have been missed; however, we prioritized the identification of toxin domains with high confidence.

In total, we identified 128 groups of effector toxins (S2 Table and Fig 1F). Eighty-three were already described in public databases (e.g., Ntox47) [9,28], or represent individual effectors that were experimentally characterized but for which HMMs have not been produced and made available (e.g., TreTu) [24,42–57]. For the latter, the newly created HMMs were named with a “.st” suffix (e.g., TreTu.st). Within the 128 candidates, 45 groups comprise new toxin domains or highly divergent variations that were not detected by previously published HMMs (Fig 1E). This justified them being distinct groups requiring the design of new HMMs. These groups of effectors were named STox followed by a number (e.g. STox_1) (S2 Table and Fig 1F). It is worth noting that these effectors are not just present in *Salmonella* and are detected widespread across several species, comprising polymorphic toxin domains [9]. Inspection of genomic context across several bacterial species revealed that most candidates exhibited a conserved adjacent gene coding for a predicted immunity protein, thus suggesting antibacterial activity (83.6%, 105/128). Some effectors, which lacked conserved immunity proteins, were predicted to display anti-eukaryotic activity (12.4%, 17/128) (S2 Table and Fig 1F). The analysis revealed a diverse array of cellular targets and biochemical activities among the 128 toxin groups (S2 Table and Fig 1F). Notably, the activities of a few STox effectors could not be predicted confidently and will require further analysis (Fig 1F). All details regarding the toxic domains identified in this study are available at https://leepbioinfo.github.io/10ksgt6ss. This includes amino acid sequence alignments, HHpred results, BLAST searches, and AlphaFold predictions. Overall, these findings highlight the significant diversity of effector toxins encoded by *S. enterica* serovars and reveal an array of novel proteins used in interbacterial competition and as virulence factors.

### *Salmonella* serovars encode unique subsets of effector toxins

**Fig 2:**
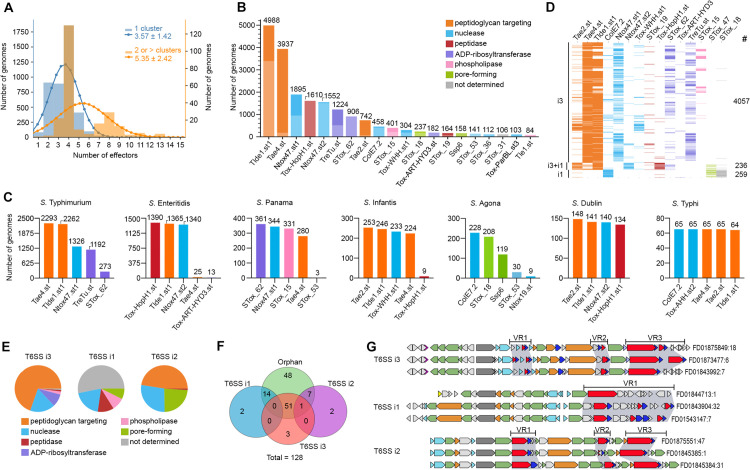
Unique subsets of effectors are associated with specific *Salmonella* serovars and T6SS subtypes. **(a)** Normal distribution and fitted curve showing the number of T6SS effectors per genome (single T6SS cluster in blue and ≥2 T6SS clusters in orange). **(b)** The most frequent effectors identified in the 10KSG dataset. Each bar represents the number of genomes encoding a specific effector. Colors represent different effector activities, with light colors representing orphan effectors while dark colors represent effectors encoded within the structural cluster. **(c)** The five most frequent effectors encoded in different *Salmonella* serovars. Colors indicate the effector activity as in (b). **(d)** Schematic representation of the most common sets of effectors in genomes encoding different T6SS subtypes. The number of genomes is indicated on the right. Colors represent activity as shown in (b). **(e)** Pie chart illustrating the relative proportions of effectors classified by activity encoded within the T6SSs subtypes i3, i1, and i2. Colors as shown in (b). **(f)** Venn diagram illustrating the proportion of overlap between effectors encoded within each T6SS structural cluster (blue: i1; purple: i2; red: i3; and green: orphan). **(g)** Schematic representation of the genetic organization of T6SSs showing the position of variable regions in which the effector and immunity proteins are encoded. Colors denote structural proteins forming the membrane complex (orange), sheath and inner tube (light blue), baseplate and spike components (green). Effectors are shown in red, and immunity proteins in dark blue. The data and code needed to generate this figure can be found in https://doi.org/10.5281/zenodo.18590644.

Genomes carrying one T6SS cluster usually encode between three and four effectors, while genomes encoding two or more T6SS clusters encoded more than 5 effectors (Fig 2A). The most frequent effectors detected within the 10KSG dataset were the peptidoglycan-targeting effectors Tlde1, a L,D-transpeptidase [23]; and Tae4, a papain-like amidase [42] (Fig 2B). These effector families were followed by Ntox47, a predicted RNase with a BECR fold [58]; the metallopeptidase Tox-HopH1; a second more divergent group of Ntox47 (Ntox47.st2); the ADP-ribosyltransferases TreTu [43] and STox_62; and another peptidoglycan-targeting effector Tae2 [42] (Fig 2B). Together, these eight effectors were found in most genomes within the 10KSG and constitute the core T6SS effectors. In addition, by analyzing the combination of effectors in each genome, we found that 88% of the genomes within the 10KSG dataset were estimated to encode up to 4 effectors per genome (mode = 3), while 12% encode combinations that range between 5–18 effectors (mode = 5) (S1 Fig).

Next, we determined the 5 most frequent effectors detected in each of the 149 *Salmonella* serovars. Serovars that predominantly encode subtype i3, such as *S.* Typhimurium. *S.* Panama, *S.* Infantis, and *S.* Typhi, harbor effectors targeting peptidoglycan (e.g., Tae4, Tlde1, Tae2), nucleases (e.g., Ntox47, Tox-WHH, ColE7, Tox-AHH), and ART enzymes (e.g., TreTu, STox_62) (Fig 2C and 2D). *S.* Dublin, which contains both subtypes i1 and i3, displays a mixture of effectors targeting the peptidoglycan (Tae2 and Tlde1), a nuclease (Ntox47), and a metallopeptidase (Tox-HopH1) (Fig 2C and 2D). *S.* Agona encodes only a T6SS subtype i1 and contains effectors with nuclease (ColE7, Ntox19, and STox_53), and pore-forming (STox_18 and STox_47) activities (Fig 2C). The core effectors of each serovar can be found in S2 Fig.

### Each *Salmonella* T6SS subtype is associated with target-specific effectors

We analyzed the combination of effectors most frequently encoded in genomes containing phylogenetically distinct T6SSs. Our findings revealed that the antibacterial subtype i3 predominantly displays a combination of peptidoglycan-targeting effectors (e.g., Tae4 and Tlde1); nucleases (e.g., Ntox47 and Tox-WHH); and ART family enzymes (e.g., TreTu and STox_62) (Fig 2D and 2E). Conversely, genomes encoding the anti-eukaryotic subtype i1 lack peptidoglycan-targeting effectors and show a combination of nucleases (e.g., ColE7), pore-forming toxins (e.g., Ssp6), and effectors with undetermined activity (Fig 2B and 2C). For genomes encoding both subtypes i1 and i3, there was a mix of effectors with antibacterial (e.g., Tae2, Tlde1, Ntox47) and anti-eukaryotic activity (e.g., Tox-HopH1) (Fig 2D and 2E). This data supports the classification of *Salmonella* T6SS subtype i3 as antibacterial and subtype i1 as anti-eukaryotic.

Notably, effectors encoded within or close to T6SS structural clusters, show minimum overlap (Fig 2F), suggesting an evolutionary scenario where T6SS clusters are acquired alongside the associated set of effectors and/or that certain subsets of effectors are preferably exchanged among bacteria harboring similar T6SS subtypes. Previous analysis of subtype i3 identified the insertion of three variable regions between the structural genes (VR1–3) in which effectors are encoded [16] (Fig 2G). Our results indicate that VR1 and VR2 contain mainly toxins targeting the periplasm (e.g., Tae2, Tae4, Tlde1, Tox-Act1) whereas VR3 primarily houses toxins targeting the cytoplasm (e.g., Ntox47, Tox-WHH, TreTu). The latter are typically associated with an N-terminal PAAR domain and Rhs (Rearrangement hotspot) repeats, which is usually fused to effectors that act on the cytoplasm [43,58–60] (Fig 2G). The position of effectors at the edge of the T6SS cluster and the domain architecture containing Rhs repeats facilitate recombination events [58], possibly leading to the greater diversity of nuclease effectors observed at the VR3 (S1 Fig).

### Tox-Act1 is a T6SS effector used for interbacterial competition in the mouse gut

**Fig 3:**
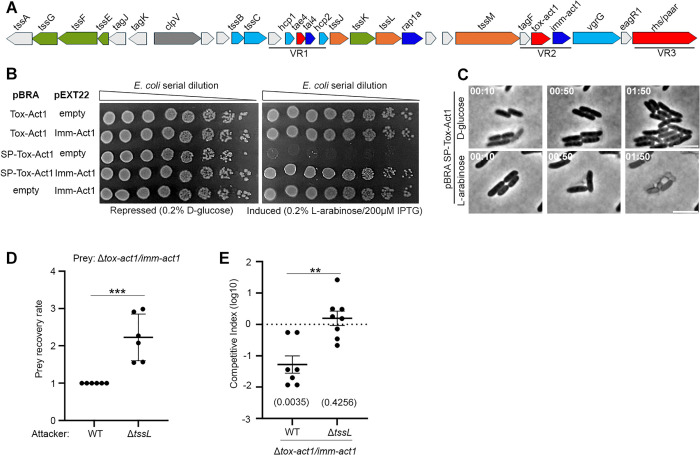
Tox-Act1 is a T6SS effector used for interbacterial competition in the mouse gut. **(a)** Scheme of the genomic region encoding Tox-Act1 and Imm-Act1 effector/immunity pair. **(b)** *E. coli* toxicity assay. Serial dilutions of *E. coli* carrying pBRA and pEXT22 constructs. Images are representative of three independent experiments. **(c)** Time-lapse microscopy of *E. coli* carrying pBRA SP-Tox-Act1 grown on repressed or induced conditions. Scale bar: 5 µm. Timestamps in hh:mm. **(d)** In vitro interbacterial competition assay using *S.* Typhimurium IC33Q strain (WT, Δ*tssL*, and Δ*tox-act1/imm-act1*). Data represents the mean ± SD of six independent experiments and were analyzed by comparison with WT that were normalized to 1. ***p < 0.001 (Student *t* test). **(e)** Groups of four C57BL/6 mice were infected by oral gavage with equal numbers of each strain. Bacteria were recovered from the cecum 4 days after infection, and competitive index (CI) values calculated. The CI is calculated as the ratio of mutant to wild-type bacteria recovered from the output normalized by their ratio in the input inoculum. The log10 CI was used for statistical analysis. Single sample *t* test was used to compare the CI to the hypothetical value of 0, and *p* value is indicated in brackets. Unpaired t *t*est (**p < 0.01) was used to compare the two groups. Dots represent individual mice from two independent experiments. The source data for this figure can be found in https://zenodo.org/records/18590644/files/10ksgt6ss-10.zip?download=1.

Among the newly identified effectors, STox_15, which was renamed Tox-Act1 (toxin acyltransferase 1, see details below), emerged as one of the most abundant (Fig 2B). To analyze whether Tox-Act1 and its associated downstream gene (Imm-Act1) form an effector and immunity pair (Fig 3A), we cloned these genes in compatible vectors under the control of different promoters and assessed toxicity upon expression in *Escherichia coli*. Tox-Act1 is toxic in the periplasm (SP-Tox-Act1) but not in the cytoplasm (Tox-Act1) of *E. coli* and co-expression with Imm-Act1 neutralizes the effect (Fig 3B).

We performed time-lapse microscopy to evaluate bacterial growth and morphology at the single-cell level. *E. coli* carrying the plasmid with SP-Tox-Act1 grew normally in D-glucose (repressed) (S1 Movie); however, shortly after induction of SP-Tox-Act1 with L-arabinose, cells began lysing (S2 Movie). It was curious that cells lysed without losing their rod shape, which suggests that the peptidoglycan was not affected (Fig 3C). After lysing, residual structures resembling the intact peptidoglycan sacculus remained (Fig 3C), indicating that this is likely not the target of Tox-Act1. In addition, we noticed that the cognate immunity protein Imm-Act1 contains a conserved domain with two transmembrane helices (S3 Fig), suggesting that the site of its neutralizing action occurs at the cell membrane.

The SPI-6 T6SS of *Salmonella enterica* serovar Typhimurium is repressed by the silencer protein H-NS [18]. However, deletion of *hns* does not fully activate the system; in our hands, it leads to only a modest increase in activity [23]. Full activation of SPI-6 T6SS occurs only under the specific conditions present in the mouse gut [19]. We found that certain *S.* Typhimurium strains, such as IC33Q (FD01843896) - which naturally encodes Tox-Act1 – express low but detectable levels of the T6SS, enabling us to perform interbacterial competition assays in vitro (Fig 3D). In these assays, prey recovery rate was higher when competing Δ*tox-act1/imm-act1* against the T6SS mutant (Δ*tssL*) than against the wild-type (WT) strain (Fig 3D), confirming that Tox-Act1 is a T6SS-dependent antibacterial effector. Although the basal expression of SPI-6 T6SS in strain IC33Q was sufficient to support interbacterial competition in vitro, it was too low to detect Tox-Act1 secretion in the culture supernatant. To assess its role in vivo, we performed competitive index assays by orally infecting mice with a 1:1 mixture of WT and Δ*tox-act1/imm-act1*, or Δ*tssL* and Δ*tox-act1/imm-act1*. The Δ*tox-act1/imm-act1* mutant showed a competitive disadvantage during colonization of the gut compared to the WT, but not when compared to Δ*tssL* (Fig 3E). These results demonstrate that Tox-Act1 is secreted via the SPI-6 T6SS and actively contributes to interbacterial competition in the mouse gut.

### Tox-Act1 is evolutionarily related to NlpC/P60 enzymes targeting lipids

The Tox-Act1 domain was not identified by any of the HMMs deployed in the initial steps of this study. However, subsequent HHpred analysis revealed a significant probability of homology with DUF4105 and the effector TseH from *Vibrio cholerae* [48] (https://leepbioinfo.github.io/10ksgt6ss/alns/STox_15.hhr.html), both of which are members of the NlpC/P60 superfamily [61,62]. This superfamily was previously defined as encompassing four families, which are divided into two higher-order groups (canonical and permuted) [61]. Members of the canonical group (AcmB-like and P60-like) function as peptidases involved in peptidoglycan hydrolysis [63], while permuted members (YaeF-like and LRAT-like) exhibit a circular permutation in their catalytic core, creating a hydrophobic binding pocket that provides specificity for lipids [64,65]. Closer inspection of the sequence and structure of Tox-Act1 revealed a circular permutation of the catalytic domain indicating that it belongs to the permuted NlpC/P60 group (https://leepbioinfo.github.io/10ksgt6ss/) [61].

**Fig 4:**
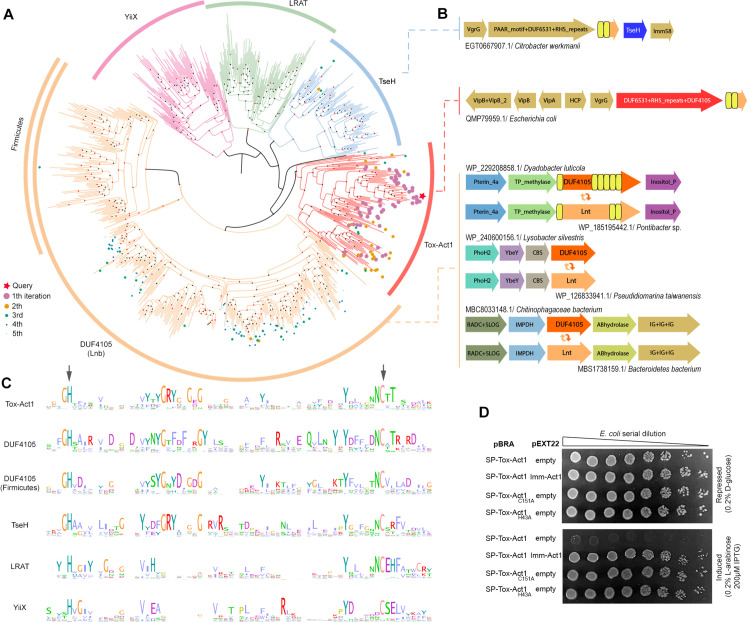
Tox-Act1 is evolutionarily related to lipid-targeting enzymes with a permuted NlpC/P60 domain. **(a)** Maximum-likelihood phylogenetic tree of permuted NlpC/P60 members. Dots represent the number of PSI–BLAST iterations required to collect homologs and the red star marks the query. **(b)** Genomic organization of representatives from clades TseH and Tox-Act1 showing the genes are encoded in the context of conflict systems, and DUF4105 showing context of lipid metabolism. **(c)** Sequence logo from the enzymatic core of permuted NlpC/P60 from all clades shown in **(a)**. The arrows indicate conserved His and Cys residues that were mutated in (d). **(d)** *E. coli* toxicity assay. Serial dilution of *E. coli* containing pBRA and pEXT22 constructs. Images are representative of three independent experiments. The source data for this figure can be found in https://zenodo.org/records/18590644/files/10ksgt6ss-10.zip?download=1.

To predict the function of Tox-Act1, we investigated its evolutionary relationship by constructing a phylogenetic tree using homologs identified through multiple PSI–BLAST searches seeded with representative sequences of Tox-Act1, TseH, LRAT, and YiiX. The resulting dataset was clustered to remove redundancy, aligned, and used to infer phylogeny, which revealed five major clades: YiiX, LRAT, TseH, Tox-Act1, and DUF4105 (Fig 4A; S3 and S4 Tables). The red star in Fig 4A marks the Tox-Act1 sequence used as the seed in its respective PSI–BLAST search, while colored dots indicate the number of iterations required to retrieve each homolog. Genomic context analysis further suggested that proteins within the Tox-Act1 clade are likely toxins deployed in biological conflicts, as they are frequently associated with antibacterial secretion systems (Fig 4A and 4B). Proteins in this clade are encoded either fused or in the vicinity of an antibacterial secretion system that has been reported to play a role in biological conflicts (e.g., T5SS, T6SS) [9]. The gene neighborhood of a Tox-Act1 homolog encoded by *E. coli* (accession# QMP79959.1) is shown as an example of this arrangement (Fig 4B). Similarly, the TseH clade (Pfam DUF6695) displays genomic contexts indicative of biological conflicts (Fig 4A and 4B). The clades LRAT and YiiX harbor proteins known to be involved in lipid metabolism: LRAT (lecithin:retinol acyltransferase) is an enzyme present in mammals and involved in the transference of acyl groups from phosphatidylcholine to all-trans retinol to produce all-trans retinyl esters that are storage forms of vitamin A [66]; H-RAS-like suppressor proteins are a group within the LRAT family that display both acyltransferase and phospholipase A1/2 activities [67]; and YiiX-like family members from *Bacillus cereus* are active against lipids [65]. We decided to name Tox-Act1 (toxin acyltransferase 1) due to its evolutionary relationship with acyltransferases.

Interestingly, Tox-Act1 emerged as the sister clade of DUF4105 (Fig 4A and 4B). Our comparative genomic analysis revealed a recurring evolutionary pattern in which DUF4105 domain-containing proteins are repeatedly displaced by apolipoprotein N-acyltransferases (Lnt) across three distinct genomic contexts (Fig 4B). Hence, we proposed that DUF4105 could be working as an acyltransferase. Remarkably, DUF4105 was recently identified as the missing lipoprotein N-acyltransferase in *Bacteroides* [68], which was named Lnb (N-acyltransferase in *Bacteroides*). These experimental results confirmed our independent *in-silico* prediction for the function of DUF4105. Given that Lnb acts on diacylated lipoproteins, its evolutionary relationship with Tox-Act1 might indicate that lipoproteins could also be potential targets of the latter. It is noteworthy that the DUF4105 clade identified in our analysis consists primarily, though not exclusively, of *Bacteroides* species, with a branch enriched in Gram-positives like *Firmicutes* (Fig 4A). The list of homologs containing DUF4105 can be found in S3 Table.

Multiple sequence alignments of each of the permuted clades including Tox-Act1 revealed the conserved catalytic His and Cys residues characteristic of the NlpC/P60 superfamily (Fig 4C) [61]. Substitution of these residues for alanine (Tox-Act1_H43A_ and Tox-Act1_C151A_) eliminated toxicity in *E. coli* (Fig 4D). Western blot analysis of HA-tagged versions confirmed that lack of toxicity is not due to absence of protein expression (S4 Fig). Together, these findings confirm that the enzymatic function of the NlpC/P60 papain-like fold domain is crucial for toxicity. Collectively, the periplasmic-acting phenotype of Tox-Act1, the presence of a membrane-associated immunity protein and the fatty acyl linkage targeting activities common in the permuted NlpC/P60 members strongly support a function for Tox-Act1 in targeting membrane lipids.

### Tox-Act1 displays phospholipase activity and changes the membrane composition of intoxicated cells

**Fig 5:**
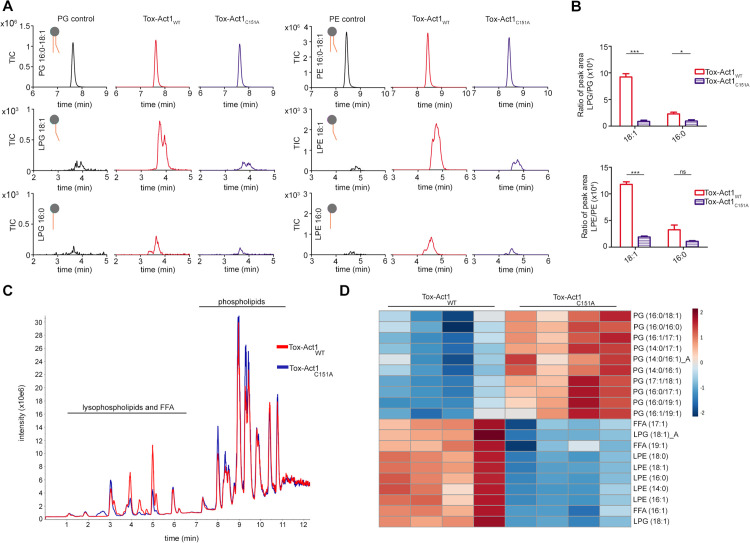
Tox-Act1 has phospholipase activity and changes the composition of target cell membranes. **(a)** In vitro enzymatic assay with recombinant Tox-Act1 (red) or Tox-Act1_C151A_ (blue) incubated with different phospholipids (PG and PE16:0-18:1). The amount of lysophospholipid produced was analyzed and quantified by HPLC-MS/MS. **(b)** Quantification of the peak area of lysophospholipids normalized by the intact substrate. Data correspond to the mean ± SD. ***p < 0.001 and *p < 0.05, *ns* not significant (unpaired *t* test). **(c)** UHPLC-MS total ion chromatogram showing the profile of total lipids extracted from *E. coli* expressing Tox-Act1 (red) or Tox-Act1_C151A_ (blue). **(d)** Heatmap plot of top 20 altered lipids of intoxicated *E. coli*. Results show four biological replicates of each condition (WT or C151A) with the quantification of lipids species (Tukey test; *p* < 0.05 FDR adjusted). Red (up) and blue (down) represents changes in lipid species concentration relative to the normalized mean. Letters differentiate between the isomers. The source data for this figure can be found in https://zenodo.org/records/18590644/files/10ksgt6ss-10.zip?download=1.

To analyze the enzymatic activity of Tox-Act1, we incubated purified recombinant protein (S5 Fig) with either purified phosphatidylglycerol (PG) 16:0–18:1 or phosphatidylethanolamine (PE) 16:0–18:1 and analyzed the reaction product by HPLC coupled to mass spectrometry (Fig 5A and 5B). Results showed that Tox-Act1 has phospholipase activity and cleaves both PG and PE (a preference for cleaving off the 16:0 acyl chain) as observed by the accumulation of 18:1 lysophospholipids (Fig 5A and 5B).

To further evaluate the enzymatic activity of Tox-Act1 and explore its functional relationship with another NlpC/P60-containing T6SS effector and a well-described phospholipase, we performed side-by-side in vitro phospholipase assays using Tox-Act1, TseH, and Tle2 from *V. cholerae*. Using our established mass spectrometry-based protocol with PG and PE substrates (16:0–18:1), we observed production of lysophospholipids by Tox-Act1, but neither TseH nor Tle2 exhibited detectable activity under these conditions (S6 and S7 Figs), at either pH 7.5 or 8.9. Intrigued by these results, we employed the fluorescence-based assay previously described for Tle2 [55], which uses a short-chain fluorescent PE analog embedded in liposomes. In this assay, Tle2 displayed strong phospholipase activity at both pH 7.5 and 8.9, while TseH showed activity only at pH 8.9. Curiously, Tox-Act1 was not active in these conditions (S8 Fig). All assays included catalytic point mutants as controls, which showed no activity, confirming the specificity of the observed reactions. These results suggest that the enzymatic activity of these effectors may be sensitive to the way the substrates are presented to these enzymes. Notably, the mass spectrometry assay uses longer-chain phospholipids solubilized in deoxycholate, which may result in solubilization of the phospholipids, while the fluorescence-based assay embeds the substrate in liposomes. These findings suggest that the physiological targets and activation contexts of these enzymes may differ. Collectively, these findings support the phospholipase activity of Tox-Act1 and TseH, reinforcing the proposed functional conservation between these effectors.

Next, we set out to determine whether Tox-Act1 could cause changes in the composition of phospholipids when ectopically expressed by target cells. *E. coli* harboring the SP-Tox-Act1 plasmid were grown to OD_600nm_ of 1.0 in the presence of D-glucose (repressed), washed, and resuspended in AB medium with L-arabinose to induce the expression of the toxin. Total lipids were extracted and analyzed by UHPLC-MS. We observed a general decrease in intact phospholipid forms in Tox-Act1_WT_, especially PG, when compared with the catalytic mutant Tox-Act1_C151A_ (Fig 5C and 5D). In addition, an increase in lysophospholipids forms—either lysophosphatidylglycerol or lysophosphatidylethanolamine—and free fatty acids (FFA) was detected in the WT (Fig 5C and 5D). The full dataset of all identified lipid species and the 23 lipid species that showed statistically significant changes can be found in S5 Table.

Lysophospholipids possess amphiphilic properties and have an inverted cone-shaped molecular structure that interacts with and modifies membrane properties (e.g., curvature) similarly to detergents [69]. The accumulation of lysophospholipids on the target cell membrane likely promotes the observed membrane disruption (Fig 3C) due to its detergent-like properties [70]. Collectively, these results confirm that Tox-Act1 targets phospholipids similarly to other proteins possessing a permuted NlpC/P60 domain, and that Tox-Act1 displays phospholipid-degrading activity. It remains to be determined whether Tox-Act1 also possesses acyltransferase activity as reported for other permuted NlpC/P60.

## Discussion

Our comprehensive analysis on the 10KSG dataset has significantly expanded the repertoire of T6SS effectors and polymorphic toxins in general, identifying 128 candidates, among which 45 comprise novel protein domains. Our study employed a robust bioinformatic pipeline, integrating classical methods with the latest structural bioinformatics techniques. The combination of sensitive sequence and structure searches with comparative genomics provided a comprehensive understanding of the identified toxin domains. In addition, the manual curation of candidates ensured high confidence in our results, distinguishing our approach from previous large-scale *in-silico* analyses. The identification of these novel toxin domains builds upon the foundational work by Zhang and colleagues [9] and others [20,33,71–78], which characterized the diversity of polymorphic toxin systems across bacterial lineages. The identification of novel toxin domains highlights the constant evolutionary arms race between bacteria, driving the diversification of toxin and immunity proteins.

This study not only broadens our understanding of *Salmonella* T6SS effectors and toxin domains but also provides the first direct characterization of a lipid-targeting NlpC/P60 toxin domain. The phospholipase activity of Tox-Act1, which cleaves acyl groups from PG and PE, underscores its role in membrane disruption during bacterial competition. Interestingly, our phylogenetic analysis revealed an evolutionary link between Tox-Act1 and a new family of lipoprotein N-acyltransferases in *Bacteroides*, adding a new dimension to the functional diversity of these toxins. Notably, one of the homologs of Tox-Act1 is TseH [48,79], which has been proposed to be an endopeptidase due to its NlpC/P60 domain and similarity to the amidase Tse1 [80,81]. However, unlike Tse1, TseH exhibits a permutation in its catalytic core [79]. This permutation, along with its evolutionary relationship to Tox-Act1 and other permuted NlpC/P60, suggest that TseH actual substrate might be an acyl group in phospholipids rather than a peptide/amide bond. Our biochemical assay supports this hypothesis, as TseH displayed phospholipase activity in a fluorescence-based assay using liposome-embedded substrates (S8 Fig). Exploring the potential acyltransferase activity of Tox-Act1 and its homologs could reveal further biochemical diversity within the NlpC/P60 superfamily.

In the context of *Salmonella* biology, the unprecedented diversity of T6SS effectors presents numerous opportunities for new studies. Our findings reveal the existence of individual subsets of T6SS effectors for each serovar, suggesting that *Salmonella* acquire and maintain effectors in response to specific environmental pressures rather than accumulating an increasingly larger array of effectors. Notably, we observed a higher number of effectors in serovars isolated from environmental sources compared to those from patients, indicating that the number of effectors increases in more diverse environments where there are potentially more encounters with a variety of rival species. In addition, we observed that *Salmonella* serovars encode phylogenetically distinct T6SS clusters, which are specialized to target either eukaryotic or bacterial cells. These results further highlight the versatility of the T6SS aiding in adaptation to many environments and hosts.

In conclusion, our comprehensive analysis has greatly enhanced the understanding of toxins involved in bacterial competition and pathogenesis. The identification of previously uncharacterized toxin domains highlights the potential for discovering novel biochemical activities. This study provides a solid foundation for future research into the complex dynamics of conflict systems and their implications for bacterial ecology and pathogenesis.

## Materials and methods

### Comparative genomic analysis

The files from the genome assemblies retrieved from the 10KSG project [26] were obtained from the authors on July 20th 2020, organized and stored in a tabular format using Python scripts, based on the Biopython [82] and pandas [83] libraries. Iterative searches were conducted using Jackhmmer [84] with a 10e−6 e-value cutoff. Protein clustering was performed using MMseqs [85] to remove redundancy (80% coverage and 70% identity) and form homologous groups (80% coverage and e-value ≤ 10e−3). Multiple sequence alignments were generated using the local-pair algorithm in MAFFT [86], and phylogenetic trees were constructed using FastTree [87]. Sequence logos were derived from sequence alignments using Jalview version 2 [88]. Domain identification and annotation was performed using HMMsearch and HMMscan [84,89] and models from the databases Pfam [28], TXSScan [27], and BastionHub [90]. Remote homology identification was performed using HHpred [36] and FoldSeek [38].

### Graph-based clustering of genomic loci

Following the removal of low-quality assemblies from the 10KSG dataset, all annotated protein sequences were clustered using MMseqs2 with 100% sequence identity and 100% coverage to eliminate redundancy. Domain annotations were then performed using HMMsearch with models from Pfam, TXSScan, BastionHub, and custom-built HMMs to identify domains associated with the T6SS components (S6 Table). To investigate the genomic context of T6SS genes, we extracted 10 genes upstream and downstream of each identified core T6SS gene, defining the genomic sites used in this study. Genomic sites located on the same contig and separated by four or fewer coding sequences were merged and assigned a unified identifier to avoid redundancy. All proteins within these loci were clustered using MMseqs2 (80% coverage, e-value ≤ 10e−3), and each resulting cluster was assigned a unique identifier. For the classification of genomic sites, each site was represented as a set of protein cluster identifiers. Pairwise similarity between genomic sites was calculated using the Jaccard index: *J*(*A*,*B*)=|*A*∩*B*|/|*A*∪*B*| where *A* and *B* are the sets of protein cluster identifiers present in each genomic site. Pairs of genomic sites with a Jaccard index >0.33 were connected by edges in a similarity graph. We then applied the Louvain community detection algorithm to this graph to identify clusters of loci with similar gene content and organization. This unsupervised approach initially grouped all genomic sites into 46 distinct communities (S1 Table). Analysis of the protein domain composition for genomic sites revealed distinctive signatures in each community. Communities enriched in tailocin and/or phage proteins could be identified by the presence of hits to Pfam models of phage tail components, while Pfam models for known T6SS components were present only at genomic sites devoid of phage tail components. We thus assigned a preliminary annotation as T6SS cluster to genomic sites of all communities without hits to phage tail components and with at least four T6SS-specific genes in 50% or more genomic sites. Similarly, communities with a minimum of three phage tail components, but without any other phage proteins, were annotated as tailocin loci. Subsequent phylogenetic analysis of conserved T6SS components (e.g., Hcp, VgrG, PAAR, baseplate subunits; see S1 Data) allowed us to consolidate these 46 communities into six broader categories: T6SS subtypes i1, i2, i3, i4B, tailocin/phages, and orphan. Once identified, tailocins and phages were removed from our dataset and not considered for further analysis. The orphan category was assigned to all genomic site communities encoding fewer than four core T6SS and less than three phage tail components in at least 50% of the genomic sites in each community (S1 Table). The requirement on the number of marker genes ensured that only genomic sites with sufficient phylogenetic signal were used for confident subtype assignments. All data and code used for sequence and genome context analyses are available on a GitHub repository at https://github.com/leepbioinfo/10ksgt6ss.

### Bacterial strains

A list of bacterial strains used in this work can be found in S7 Table. Strains were grown at 37°C in Lysogeny Broth (10 g/L tryptone, 10 g/L NaCl, 5 g/L yeast extract) under agitation. AB medium was used for lipidomics: 0.2% (NH4)_2_SO_4_, 0.6% Na_2_HPO_4_, 0.3% KH_2_PO_4_, 0.3% NaCl, 0.1 mM CaCl_2_, 1 mM MgCl_2_, 3 μM FeCl_3_, supplemented with 0.2% sucrose, 0.2% casamino acids, 10 μg/mL thiamine, and 25 μg/mL uracil. Cultures were supplemented with antibiotics in the following concentration when necessary: 50 μg/mL kanamycin, 100 μg/mL ampicillin, and 50 μg/mL streptomycin.

### Cloning and mutagenesis

All primers are listed in S7 Table. Tox-Act1 and Imm-Act1 were amplified by PCR and cloned into pBRA vector under the control of P_BAD_ promoter [91] with or without pelB signal peptide sequence from pET22b (Novagen) [92]. Imm-Act1 was cloned into pEXT22 under the control of P_TAC_ promoter [93]. For protein expression and purification, Tox-Act1 was cloned into pET28a (Novagen), including a C-terminal Strep II tag. Point mutations (Tox-Act1_H43A_, Tox-Act1_C151A_) were created using QuikChange II XL Site-Directed Mutagenesis Kit (Agilent Technologies) or by splicing by overlap extension (SOE) PCR. All constructs were confirmed by sequencing. *S*. Typhimurium mutant strains used for competition assays were constructed by λ-Red recombination system using a one-step inactivation procedure [94].

### *E. coli* toxicity assay

Overnight cultures of *E. coli* DH5α co-expressing effectors for cytoplasmic (pBRA Tox-Act1) or periplasmic (pBRA SP-Tox-Act1) localization and immunity protein (pEXT22 Imm-Act1) were adjusted to OD_600nm_ 1, serially diluted in LB (1:4) and 5 μL were spotted onto LB agar (1.5%) containing either 0.2% D-glucose or 0.2% L-arabinose plus 200 μM IPTG, supplemented with streptomycin and kanamycin, and incubated at 37°C for 20 h.

### Time-lapse microscopy

For time-lapse microscopy, LB agar (1.5%) pads were prepared by cutting a rectangular piece out of a double-sided adhesive tape, which was taped onto a microscopy slide as described previously [92]. *E. coli* DH5α harboring pBRA SP-Tox-Act1 was subcultured in LB (1:50) with 0.2% D-glucose until reaching OD_600nm_ 0.4–0.6 and adjusted to OD_600nm_ 1. Cultures were spotted onto LB agar pads supplemented either with 0.2% D-glucose or 0.2% L-arabinose plus antibiotics. Images were acquired every 15 min for 16 h using a Leica DMi-8 epifluorescence microscope fitted with a DFC365 FX camera (Leica) and Plan-Apochomat ×63 oil objective (HC PL APO ×63/1.4 Oil ph3 objective Leica). Images were analyzed using FIJI software [95].

### Competitive index

Female C57BL/6 mice (6–8 weeks old) were purchased from Jackson’s Laboratory. Mice were housed in pathogenic-free conditions with unlimited access to food and water, except for 4 hours prior oral gavage. Mice were pre-treated with 20 mg of streptomycin 24 hours prior infection with *Salmonella*. Mice were infected by oral gavage with a 1:1 mixture (total of 10^10^ CFUs) of *S.* Typhimurium WT and Δ*tox-act1/imm-act1* (Cm^R^), or a mixture of Δ*tssL* and Δ*tox-act1/imm-act1*. Mice were euthanized by exposure to carbon dioxide (CO_2_) four days after infection, and cecum were harvested and the content serial diluted in PBS 1× (Phosphate-buffered saline) and plated in MacConkey Agar to determine the total CFU counts. One hundred colonies from each mouse were patched on chloramphenicol plates to determine the proportion of each strain in the mixture. The same procedure was performed with the initial mixture prior infection. Competitive index was calculated as previously described [96]. The unpaired *t* test was used to compare the CI between WT and Δ*tssL* groups, while the single-sample *t* test was used to compare each log10 CI to the hypothetical value of 0 (the value of 0 means that two strains grew equally well in vivo).

### Bacterial competition

Bacterial competition assays were performed using *S.* Typhimurium IC33Q strain, which was freshly thawed prior to each experiment. Overnight cultures of attacker and prey cells were subculture (1:50) until reaching OD_600nm_ 0.6, cultures were mixed 4:1 attacker:prey (OD_600nm_ 0.5), 5 μL spotted onto 0.22 μm nitrocellulose membranes (1 × 1 cm), and incubated on LB agar for 20 h at 37°C. The membranes containing the bacterial mixture were placed on 1.5 mL tubes containing 1 mL LB, homogenized by vortex, serially diluted, and plated on selective plates with antibiotics. Prey recovery rate was calculated by dividing the CFU counts of the output by the input. Data represents the mean ± SD of six independent experiments and were analyzed through comparison with WT that were normalized to 1.

### Protein expression and purification

*E. coli* BL21(DE3) carrying pET28a Tox-Act1_WT_-Strep or Tox-Act1_C151A_-Strep were grown in 4 L of LB supplemented with kanamycin (37°C, 180 rpm) until OD_600nm_ 0.7. Expression was induced with 1 mM IPTG for 16 h at 16°C. Cells were harvested via centrifugation at 5,000 *g* for 20 min, and pellets were resuspended in lysis buffer (50 mM Tris-HCl pH 8.0, 350 mM NaCl, 45 mM β-mercaptoethanol, 5 mg/mL lysozyme, 10% glycerol) and lysed at 4°C using a sonicator. The lysate was centrifuged at 40,000 *g* for 45 min at 4°C. The supernatant was loaded onto a 1 mL StrepTrap HP column (Cytiva) equilibrated in buffer (50 mM Tris-HCl pH 8.0, 350 mM NaCl, 10% glycerol). The column was washed with 40 column volumes (CV) of wash buffer (50 mM Tris-HCl pH 8.0, 1 M NaCl, 10% glycerol, 45 mM β-mercaptoethanol), followed by a second wash with 12 CV (50 mM Tris-HCl pH 8.0, 1.5 M urea) to remove chaperonin GroEL [97]. The column was subjected to a third round of washes with 40 CV of wash buffer and eluted with 10 CV of elution buffer (50 mM Tris-HCl pH 8.0; 350 mM NaCl; 10% glycerol; 50 mM biotin). Fractions were buffer exchanged (25 mM Tris-HCl pH 7.5, 100 mM NaCl, 5% glycerol) and concentrated using an Amicon of 30 kDa (Sigma). Protein aliquots were snap-frozen until use.

For purification of TseH (VCA0285) [79] and Tle2 (Tle2^VC^, VC1418) [55] proteins, *E. coli* BL21(DE3) carrying pET28a 6xHis-TseH_WT_ or 6xHis-TseH_C186A_ (Km^R^), and pMALC2H10 MBP-10xHis-Tle2_WT_ or MBP-10xHis-Tle2_S371A_ (Amp^R^) were subcultured in 1 L of LB supplemented with kanamycin or ampicillin (37°C, 180 rpm) until OD600_nm_ 0.7. Expression was induced with 1 mM IPTG for 16 h at 16°C. Cells were harvested via centrifugation at 5,000 *g* for 20 min, and pellets were resuspended in lysis buffer (50 mM Tris-HCl pH 8.0, 350 mM NaCl, 10 mM Imidazole, 5 mg/mL lysozyme, 5% glycerol) and lysed at 4°C using a sonicator. The lysate was centrifuged at 40,000 *g* for 45 min at 4°C. The supernatant was loaded onto a 1 mL HisTrap HP column (Cytiva) equilibrated in buffer A (50 mM Tris-HCl pH 8.0, 350 mM NaCl, 10 mM Imidazole, 5% glycerol). Column was washed with 30CV of buffer A, and elution perfomed stepwise with 3CV of 5%, 10%, 15%, 20%, 25%, 50%, and 100% of buffer B (50 mM Tris-HCl pH 8.0, 350 mM NaCl, 500 mM Imidazole, 5% glycerol). Fractions were buffer exchanged (25 mM Tris-HCl pH 7.5, 100 mM NaCl, 5% glycerol) and concentrated using an Amicon of 30 kDa (Sigma). Protein aliquots were snap-frozen until use.

### In vitro phospholipase assays

For in vitro enzymatic assays, we used two distinct approaches. The first was mass-spectrometry-based and the second fluorescence-based. For MS analysis, phospholipids 1-palmitoyl-2-oleoyl-sn-glycero-3-phospho-(1′-rac-glycerol) (PG 16:0–18:1) and 1-palmitoyl-2-oleoyl-sn-glycero-3-phosphoethanolamine (PE 16:0–18:1) were purchased from Avanti Polar Lipids. Substrates were resuspended and diluted in methanol to adjust the concentration of aliquots. The methanol of each aliquot was dried under a nitrogen flow. A total of 1.2 mM of phospholipids (PG or PE) were incubated with 800 nM of either Tox-Act1, TseH and Tle2, or their respective catalytic mutants, in reaction buffer (25 mM Tris-HCl pH 7.5, 100 mM NaCl, 0.5 mM CaCl_2_, 0.5 mM MgCl_2_, 18 mM sodium deoxycholate) in a total volume of 100 µL for 2 h at 37°C under agitation (350 rpm). For Tox-Act1 and TseH, 0.5 mM DTT was added to the reaction buffer. Lipids were extracted by adding 830 µL of a mixture of MTBE/methanol/water (10:3:2.5, v/v/v), followed by incubation under agitation for 1 h at room temperature. Samples were centrifuged for 2 min at 220 *g* and 350 µL of the top fraction was transferred to a new tube, dried in a SpeedVac, and stored at −80°C until analysis.

For mass spectrometry analysis, samples were resuspended in 350 µL of isopropanol and analyzed in a Shimadzu 8060 Triple Quadrupole Liquid Chromatograph Mass Spectrometer. Samples (0.1–0.5 µL) were loaded into an Agilent column C18 ZORBAX Eclipse Plus (4.6 × 150 mm, 5 µm, 400 bar) with a flow rate of 0.5 mL/min and an oven temperature of 40°C. HPLC gradients were as described below for lipidomic analysis. The phospholipids and lysophospholipids of interest were analyzed in the multiple reaction monitoring (MRM) mode using *m*/*z* transitions, collision energies, and dwell times as shown in S5 Table. Data was acquired by Shimadzu LabSolutions and processed in LabSolutions Browser. Graphs were plotted using GraphPrism 5.

Fluorescence-based assays were performed using liposomes embedded with the fluorescent substrate PED-A1 (ThermoFisher, Cat# A10070), following the manufacturer’s instructions, with minor modifications. Lipids were prepared by making a 10 mM solutions of PG 16:0–18:1 and of PE 16:0–18:1 dissolved in 100% ethanol. PED-A1 was stored at 5 mM in DMSO and diluted to 1 mM in ethanol prior to use. A concentrated lipid mixture was prepared by combining 10 µL each of PE (10 mM), PG (10 mM), and PED-A1 (1 mM), to a final 30 µL. Liposomes were formed by mixing 25 µL of the concentrated lipid mixture with 1.6 mL of buffer (50 mM Tris-Cl, pH 7.5 or 8.9, 100 mM NaCl, 1 mM CaCl₂) under agitation with a magnetic stir bar. For enzymatic assays, 50 µL of the liposomes containing PED-A1 were mixed with 50 µL of enzyme solution in a 96-well plate. Final reaction conditions included 800 nM Tox-Act or TseH, and 600 nM Tle2 in buffer containing 50 mM Tris-Cl (pH 7.5 or 8.9), 100 mM NaCl, 1 mM CaCl₂, 0.5 mM MgCl₂, and 0.5 mM DTT (for Tox-Act and TseH). Fluorescence was monitored at 37°C for 1 h using a Biotek Synergy H1 plate reader (Agilent), with excitation at 488 nm and emission at 530 nm.

### Lipidomics of Tox-Act1-intoxicated *E. coli*

*E. coli* MG1655 containing the plasmids pBRA SP-Tox-Act1_WT_ or SP-Tox-Act1_C151A_ were cultured in LB containing 0.2% D-glucose at 37°C for 14 h. Cells were subcultured in LB 0.2% D-glucose until an OD_600nm_ of 1 and centrifuged (10 min, 2,900 *g*, 30°C). Cells were washed with 40 mL of preheated AB medium at 37°C, centrifuged (10 min, 2,900 *g*, 30°C), and resuspended in 5 mL of AB supplemented with 0.2% L-arabinose to induce Tox-Act1 expression. Cells were incubated for 1 h at 37°C with agitation (100 rpm). The cells were centrifuged (15 min, 2,900 *g*, 4°C) and washed once with 1 mL of PBS pH 7.4. PBS was removed by centrifugation, and the cell pellet was stored at −80°C until lipid extraction. A cocktail of class-specific internal standards was added to the cell mass prior to lipid extraction for subsequent quantification and normalization (S5 Table). Total lipid extraction was performed using an adapted version of a protocol described previously [98]. Briefly, cell pellets were resuspended with 500 µL of cold methanol and 1 mL of MilliQ water and transferred to glass tubes. A mixture of chloroform and ethyl acetate (4:1) was added, followed by agitation for 1 min at 25°C. Samples were centrifuged (2 min, 1,500 *g*, 4°C) and the lipid-containing phase (lower phase) was extracted and transferred to a new glass tube that was dried under a nitrogen (N_2_) flow until all solvent traces were evaporated. Samples were stored at −80°C until analysis.

Lipid extracts were diluted in 100 µL of isopropanol and analyzed using ultra-high-performance liquid chromatography (UHPLC Nexera, Shimadzu) coupled with an ESI-Q-TOF mass spectrometer (Triple TOF 6600, Sciex) (UHPLC-Q-TOF/MS). 2 μL of each sample were injected into the UHPLC-MS, and molecules were separated using a CORTECS column (C18, 1.6 μm, 2.1 × 100 mm, Waters) with a flow rate of 0.2 mL/min and temperature set to 35°C [99]. The mobile phases consisted of (A) water/acetonitrile (60:40) and (B) isopropanol/acetonitrile/water (88:10:2). Ammonium acetate at a final concentration of 10 mM was incorporated in both mobile phases A and B for the negative ionization acquisition mode. The gradient elution used in the chromatography was from 40% to 100% (mobile phase) B over the first 10 min; 100% B from 10 to 12 min; 100% to 40% B for 12–13 and holding 40% B for 13–20 min. The negative mode was utilized for the examination of phospholipids and FFA. MS and MS/MS data acquisition was performed using Analyst 1.7.1 software (Sciex). Mass spectrometry data was inspected using PeakView 2.0 software (Sciex), and lipid molecular species were manually identified with the help of an in-house manufactured Excel-based macro. Lipid species were quantified using MultiQuant software (Sciex), where the precursor ions areas were normalized by the internal standards for each class (S5 Table).

## Ethics statement

The animal experiments were performed with protocols approved by the University of Texas at Austin, Institutional Animal Care and Use Committee (protocol #AUP-2024-00232). The University of Texas at Austin animal management program is accredited by the Association for the Assessment and Accreditation of Laboratory Animal Care, International (AAALAC), and meets National Institutes of Health standards as set forth in the Guide for the Care and Use of Laboratory Animals (DHHS Publication No. (NIH) 85–23 Revised 1996).

## Supporting information

S1 Fig T6SS effector repertoire in the 10KSG dataset. Each column indicates the presence or absence of a toxin as identified by the models developed in this study. Lines denote unique combinations of effectors. The histogram on the right shows the frequency of genomes in the 10KSG dataset containing each specific repertoire. The histogram at the bottom illustrates the frequency of genomes with at least one protein identified by the above model. Effector activities are color-coded as described in Fig 2B. The data and code needed to generate this figure can be found in https://doi.org/10.5281/zenodo.18590644.
(TIF)

S2 Fig List of five most frequent T6SS effectors identified in each of the 149 *Salmonella* serovars contained in the 10KSG dataset. Colors indicate the effector activity as in Fig 2B. The data and code needed to generate this figure can be found in https://doi.org/10.5281/zenodo.18590644.
(PDF)

S3 Fig Imm-Act1 has two transmembrane helices. **(A)** Sequence alignment of Imm-Act1 homologs with orange rectangles indicating predicted transmembrane helices. Sequences are colored according to the Clustal X color scheme [100]. **(B)** Transmembrane helix prediction for Imm-Act1 using DeepTMHMM [101]. The source data for this figure can be found in https://zenodo.org/records/18590644/files/10ksgt6ss-10.zip?download=1.
(TIF)

S4 Fig Western blot of HA-tagged Tox-Act1 versions used for toxicity assay. Expression levels of HA-tagged Tox-Act1 protein variants shown in Fig 4D. Blots were probed with anti-HA antibody to confirm protein expression prior to toxicity assays. Anti-DNAk was used as a loading control. The source data for this figure can be found in https://zenodo.org/records/18590644/files/10ksgt6ss-10.zip?download=1.
(TIF)

S5 Fig Purification of recombinant proteins used in enzymatic assays. SDS-PAGE of recombinant proteins during purification. Affinity chromatography using Strep-Tactin Sepharose to purify Tox-Act1 versions with C-terminal Strep-tag II: WT in **(A)** or C151A in **(B)**. Recombinant proteins were purified from the soluble fraction. An additional step of washing with 1.5 M urea was performed after the traditional washes to remove contamination with GroEL before elution with biotin. Additional bands were identified by mass-spectrometry to confirm identity. Purified recombinant proteins used in the enzymatic assays: **(C)** Tox-Act1 and C151A mutant; **(D)** TseH and C156A mutant; **(E)** Tle2 and S371A mutant. INS, insoluble; SOL, soluble; FT, flow through. GroEL, chaperonin GroEL; Bccp, biotin carboxyl carrier protein; OmpF, outer membrane porin F. The source data for this figure can be found in https://zenodo.org/records/18590644/files/10ksgt6ss-10.zip?download=1.
(TIF)

S6 Fig Mass-spectrometry based phospholipase assay with PG substrate. In vitro enzymatic assay with recombinant Tox-Act1, TseH, Tle2 (red) and their respective catalytic mutants Tox-Act1_C151A_, TseH_C186A_ and Tle2_S371A_ (blue) incubated with 16:0–18:1 PG at pH 7.5 **(a)** or pH 8.9 **(b)**. The amount of lysophospholipids produced was analyzed by HPLC-MS/MS. **(c–f)** Quantification of the peak area of lysophospholipids was normalized by the intact substrate. Data corresponds to the mean ± SD. *p < 0.05, *ns* not significant (Student *t* test). The source data for this figure can be found in https://zenodo.org/records/18590644/files/10ksgt6ss-10.zip?download=1.
(TIF)

S7 Fig Mass-spectrometry based phospholipase assay with PE substrate. In vitro enzymatic assay with recombinant Tox-Act1, TseH, Tle2 (red) and their respective catalytic mutants Tox-Act1_C151A_, TseH_C186A_ and Tle2_S371A_ (blue) incubated with 16:0–18:1 PE at pH 7.5 **(a)** or pH 8.9 **(b)**. The amount of lysophospholipids produced was analyzed by HPLC-MS/MS. **(c–f)** Quantification of the peak area of lysophospholipids was normalized by the intact substrate. Data corresponds to the mean ± SD. *p < 0.05, *ns* not significant (Student *t* test). The source data for this figure can be found in https://zenodo.org/records/18590644/files/10ksgt6ss-10.zip?download=1.
(TIF)

S8 Fig Fluorescence-based phospholipase assay with PE substrate. Liposomes containing the fluorescent substrate PED-A1 were incubated with recombinant Tox-Act1, TseH, Tle2 (red) or their catalytic mutants Tox-Act1_C151A_, TseH_C186A_ and Tle2_S371A_ (blue). Fluorescence is represented in arbitrary units (A.U). Data is the mean ± SD of at least four independent experiments. *p < 0.05 (Student *t* test). The source data for this figure can be found in https://zenodo.org/records/18590644/files/10ksgt6ss-10.zip?download=1.
(TIF)

S1 Table Frequency of hits to HMM models associated with T6SS and/or phages/tailocins. Each row corresponds to a group of genomic loci defined by unsupervised community detection. The first columns report the final classification after manual inspection, along with summary statistics including the number of genomes and loci contained in each unsupervised community. The next three columns show the number of profile markers found in more than 50% of the loci within each community. The remaining columns correspond to individual HMMs and indicate the percentage of loci from each classification that match the given HMM. Color code: dark green, HMMs with hits in T6SS, phages, and/or tailocins (not used for system classification); blue, T6SS-specific markers; light green, phage tail components; red, other phage components; gray, ATPases (not used for system classification). The data and code needed to generate this table can be found in https://doi.org/10.5281/zenodo.18590644.
(XLSX)

S2 Table List of all T6SS toxin domains identified in this study in the 10K *Salmonella* Genomes dataset. (XLSX)

S3 Table List of homologs included in the phylogenetic analysis presented in Fig 4A. The table includes sequence identifiers, genomic coordinates, source genomes, and relevant annotation features. The data and code needed to generate this table can be found in https://doi.org/10.5281/zenodo.18590644.
(XLSX)

S4 Table Genomic context of each homolog used to construct the phylogenetic tree shown in Fig 4A. The table includes genomic coordinates, neighboring genes, and relevant annotation features. The data and code needed to generate this table can be found in https://doi.org/10.5281/zenodo.18590644.
(XLSX)

S5 Table Full dataset of identified lipids in *E. coli* expressing Tox-Act1_WT_ or Tox-Act1_C151A_. **(A)** MRM transition for LC-MS/MS method of phospholipids and lysophospholipids. **(B)** Internal standard used for lipidomics analysis in negative mode. **(C)** Statistically altered lipids between *E. coli* expressing Tox-Act1_WT_ and Tox-Act1_C151A_. **(D)** Full dataset of identified lipids in *E. coli* expressing Tox-Act1_WT_ or Tox-Act1_C151A_.
(XLSX)

S6 Table List of T6SS markers. The Source column indicates the origin of each HMM (PFAM database; TXSSscan framework; or in-house profiles). The Model column specifies the profile name, the Replace column indicates the updated profile name, and the Marker column denotes whether the model was used as a T6SS marker (1 = yes; 0 = no). The data and code needed to generate this table can be found in https://doi.org/10.5281/zenodo.18590644.
(XLSX)

S7 Table List of strains, plasmids, antibodies and primers used in this study. (XLSX)

S1 Movie Time-lapse microscopy of *E. coli* harboring pBRA SP-Tox-Act1 growing in media supplemented with 0.2% D-glucose. Timestamp in hh:mm. Scale bar: 5 μm.
(AVI)

S2 Movie Time-lapse microscopy of *E. coli* harboring pBRA SP-Tox-Act1 growing in media supplemented with 0.2% L-arabinose. Timestamp in hh:mm. Scale bar: 5 μm.
(AVI)

S1 Data Phylogeny of T6SS components. Phylogenetic trees are shown for individual T6SS components. Blue labels indicate sequences originating from the SecReT6 dataset [34], with labels showing the classification reported in that dataset. Red labels indicate sequences derived from the 10KSG project, with labels showing the community assignments generated using the Louvain algorithm.
(PDF)

S1 Raw Images Original gel/blot images shown in S4 and S5 Figs. (TIF)

## Abbreviations

CV: column volumes
FFA: free fatty acids
HMMs: hidden Markov models
MRM: multiple reaction monitoring
PE: phosphatidylethanolamine
PG: phosphatidylglycerol
T6SS: Type VI Secretion System
WT: wild-type
