# Supplementary figures and images for: Systematic identification of Salmonella T6SS effectors uncovers diverse new families and lipid-targeting activities
Source: PLoS Biol. 2026 Mar 17;24(3):e3003680. doi: 10.1371/journal.pbio.3003680 (PMC12994826; doi:10.1371/journal.pbio.3003680)

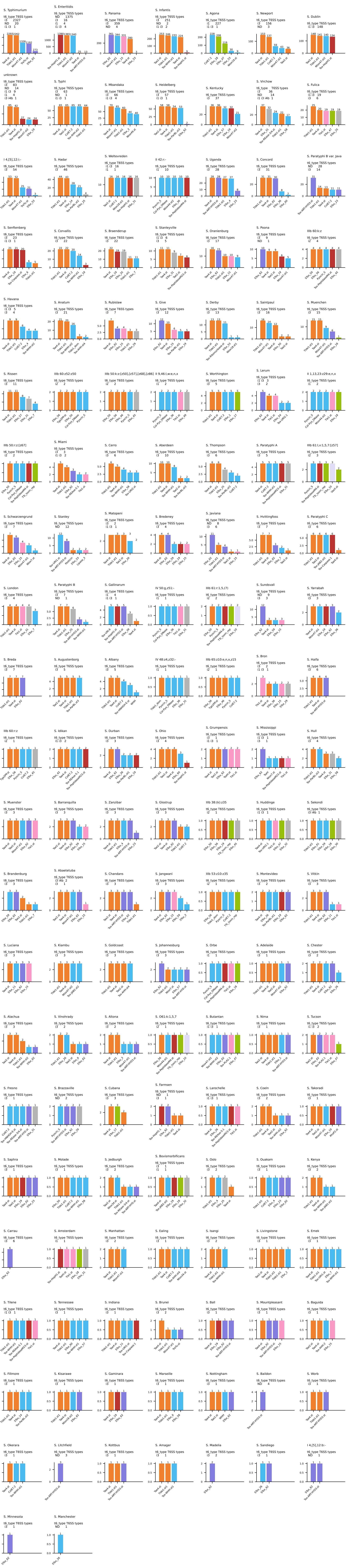

Supplement: S2 Fig — Colors indicate the effector activity as in Fig 2B. The data and code needed to generate this figure can be found in https://doi.org/10.5281/zenodo.18590644. (PDF) [file pbio.3003680.s002.pdf]

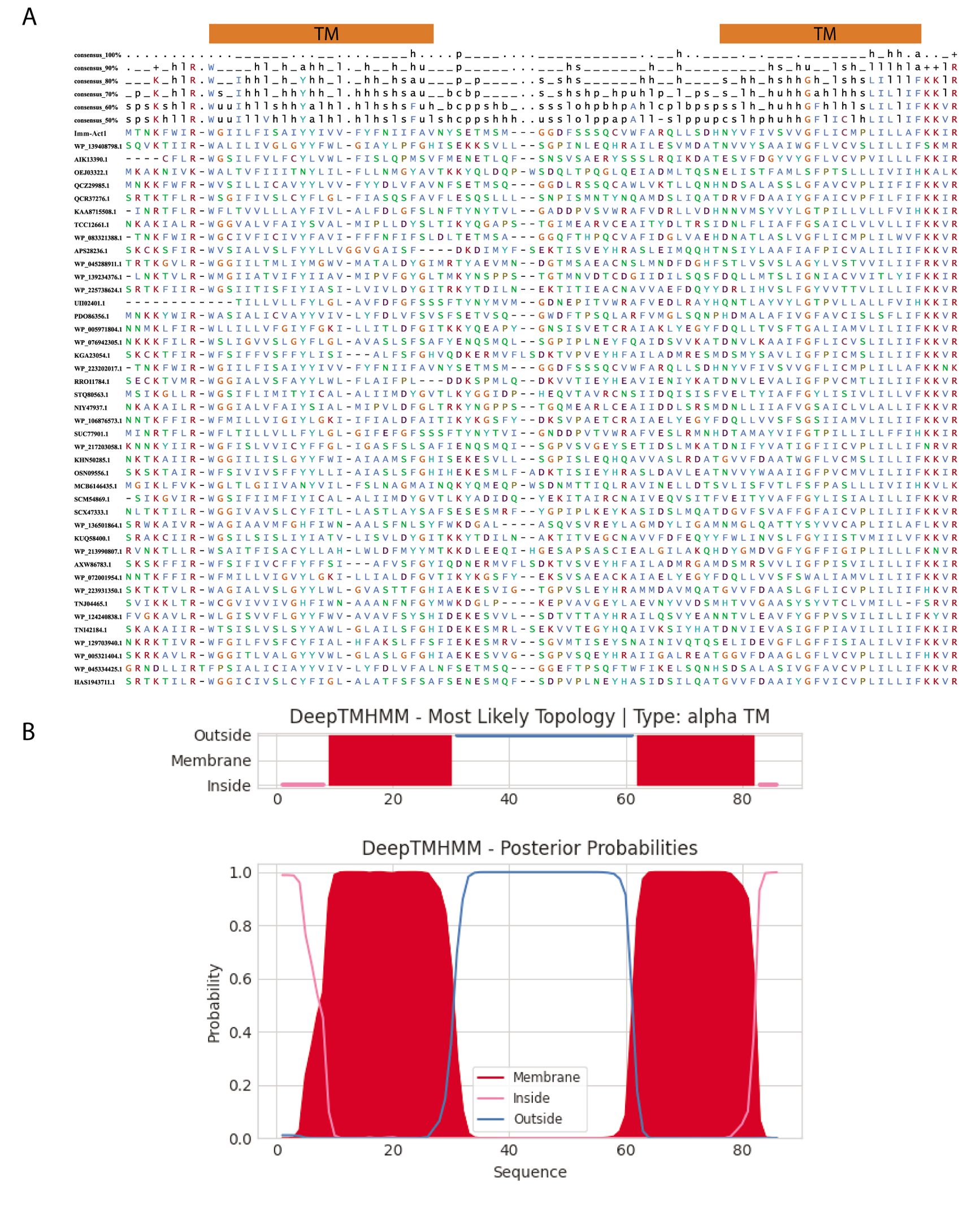

Supplement: S3 Fig — (A) Sequence alignment of Imm-Act1 homologs with orange rectangles indicating predicted transmembrane helices. Sequences are colored according to the Clustal X color scheme [100]. (B) Transmembrane helix prediction for Imm-Act1 using DeepTMHMM [101]. The source data for this figure can be found in https://zenodo.org/records/18590644/files/10ksgt6ss-10.zip?download=1. (TIF) [file pbio.3003680.s003.tif]

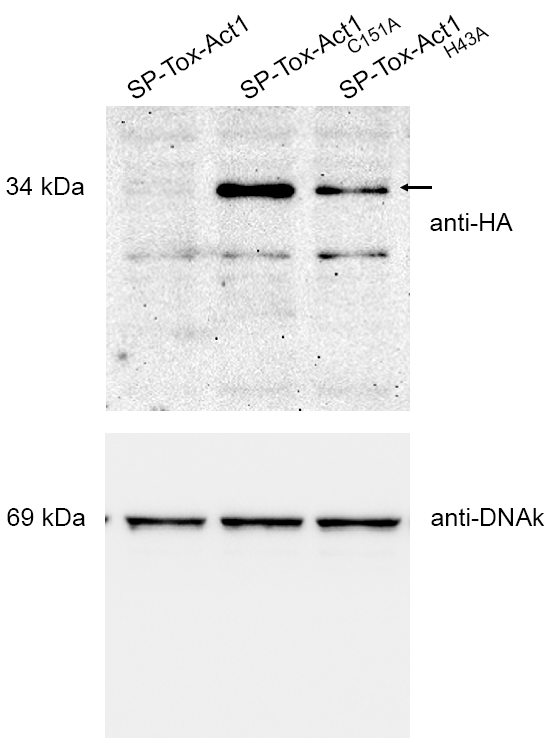

Supplement: S4 Fig — Expression levels of HA-tagged Tox-Act1 protein variants shown in Fig 4D. Blots were probed with anti-HA antibody to confirm protein expression prior to toxicity assays. Anti-DNAk was used as a loading control. The source data for this figure can be found in https://zenodo.org/records/18590644/files/10ksgt6ss-10.zip?download=1. (TIF) [file pbio.3003680.s004.tif]

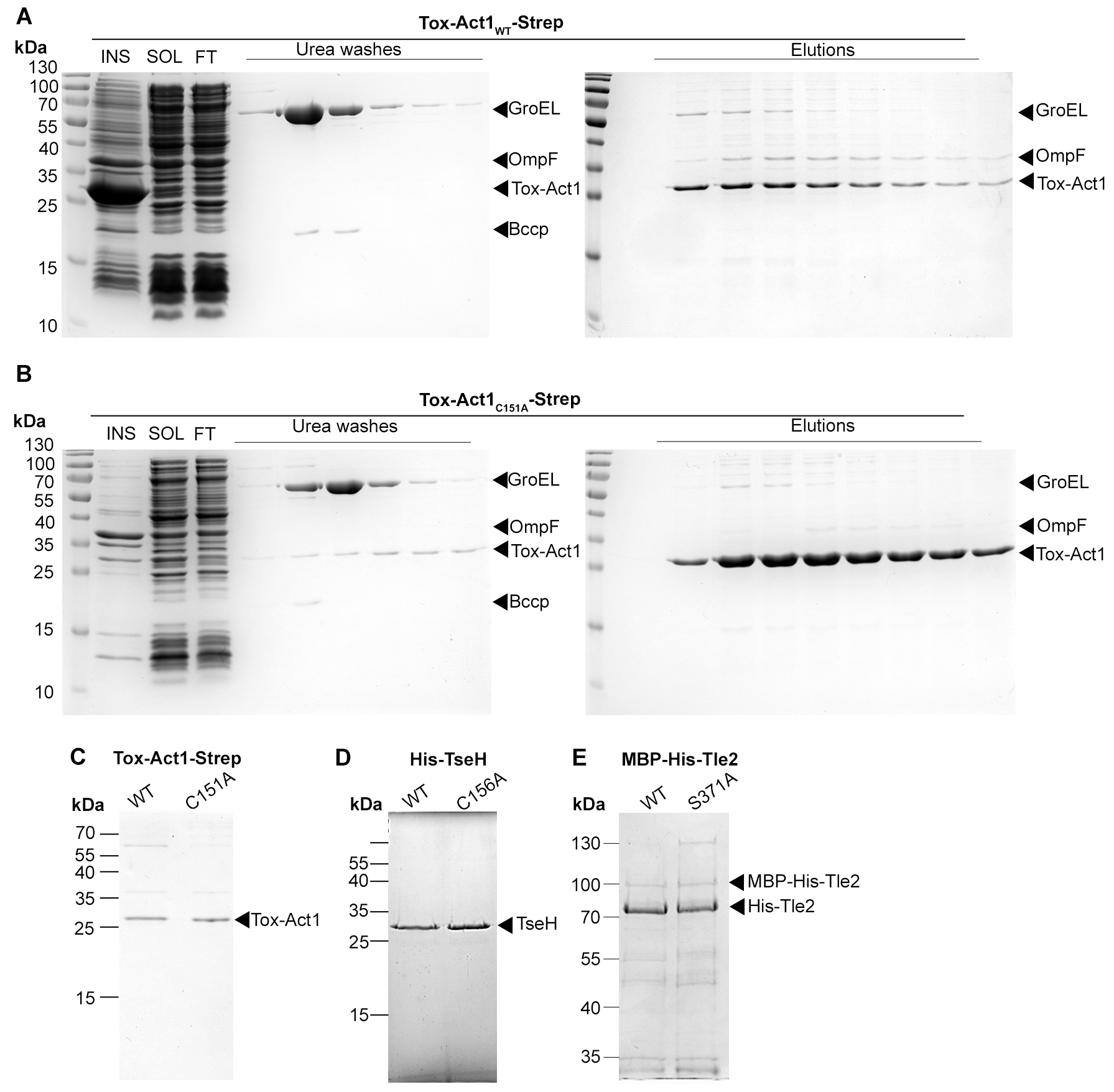

Supplement: S5 Fig — SDS-PAGE of recombinant proteins during purification. Affinity chromatography using Strep-Tactin Sepharose to purify Tox-Act1 versions with C-terminal Strep-tag II: WT in (A) or C151A in (B). Recombinant proteins were purified from the soluble fraction. An additional step of washing with 1.5 M urea was performed after the traditional washes to remove contamination with GroEL before elution with biotin. Additional bands were identified by mass-spectrometry to confirm identity. Purified recombinant proteins used in the enzymatic assays: (C) Tox-Act1 and C151A mutant; (D) TseH and C156A mutant; (E) Tle2 and S371A mutant. INS, insoluble; SOL, soluble; FT, flow through. GroEL, chaperonin GroEL; Bccp, biotin carboxyl carrier protein; OmpF, outer membrane porin F. The source data for this figure can be found in https://zenodo.org/records/18590644/files/10ksgt6ss-10.zip?download=1. (TIF) [file pbio.3003680.s005.tif]

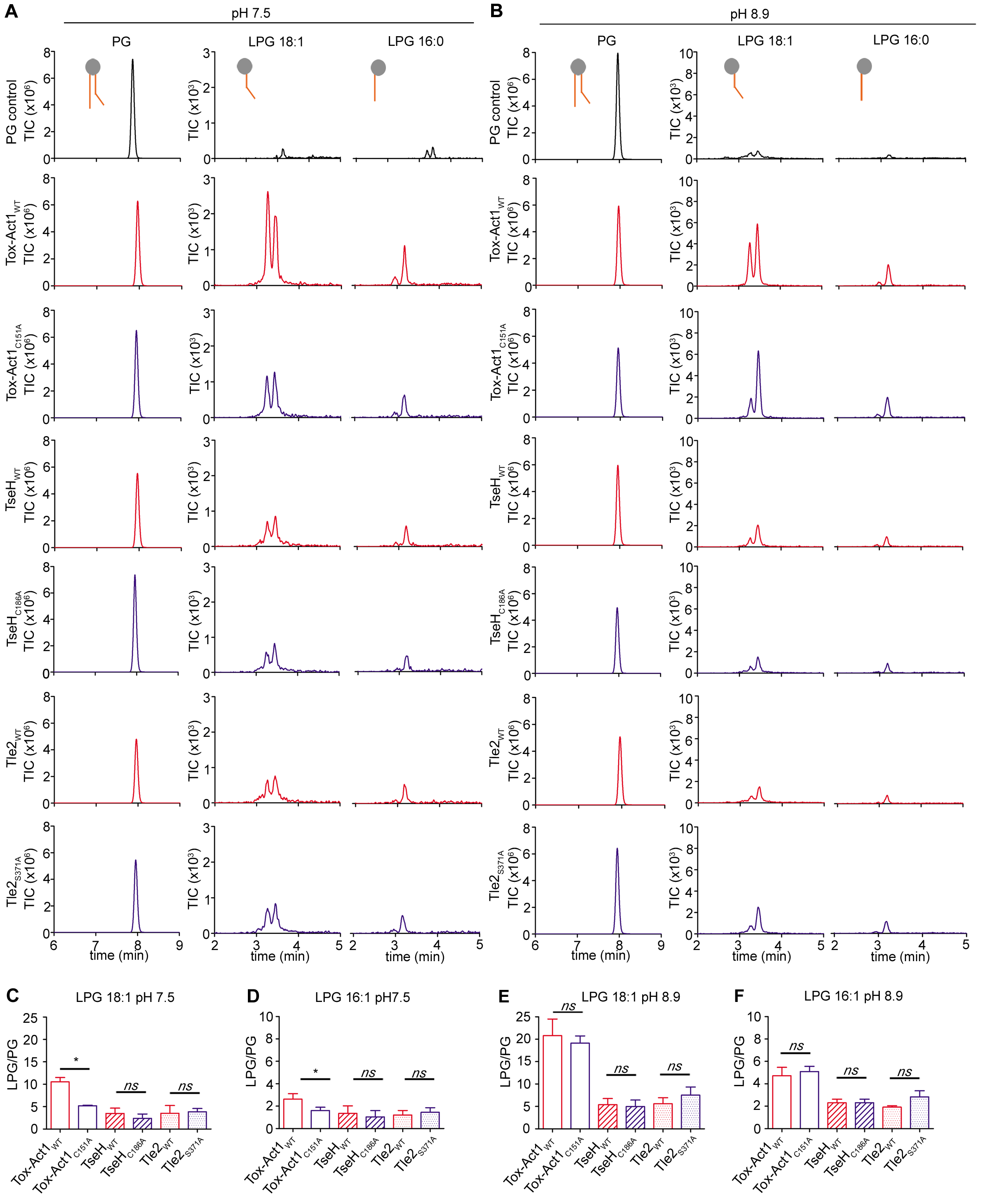

Supplement: S6 Fig — In vitro enzymatic assay with recombinant Tox-Act1, TseH, Tle2 (red) and their respective catalytic mutants Tox-Act1C151A, TseHC186A and Tle2S371A (blue) incubated with 16:0–18:1 PG at pH 7.5 (a) or pH 8.9 (b). The amount of lysophospholipids produced was analyzed by HPLC-MS/MS. (c–f) Quantification of the peak area of lysophospholipids was normalized by the intact substrate. Data corresponds to the mean ± SD. *p < 0.05, ns not significant (Student t test). The source data for this figure can be found in https://zenodo.org/records/18590644/files/10ksgt6ss-10.zip?download=1. (TIF) [file pbio.3003680.s006.tif]

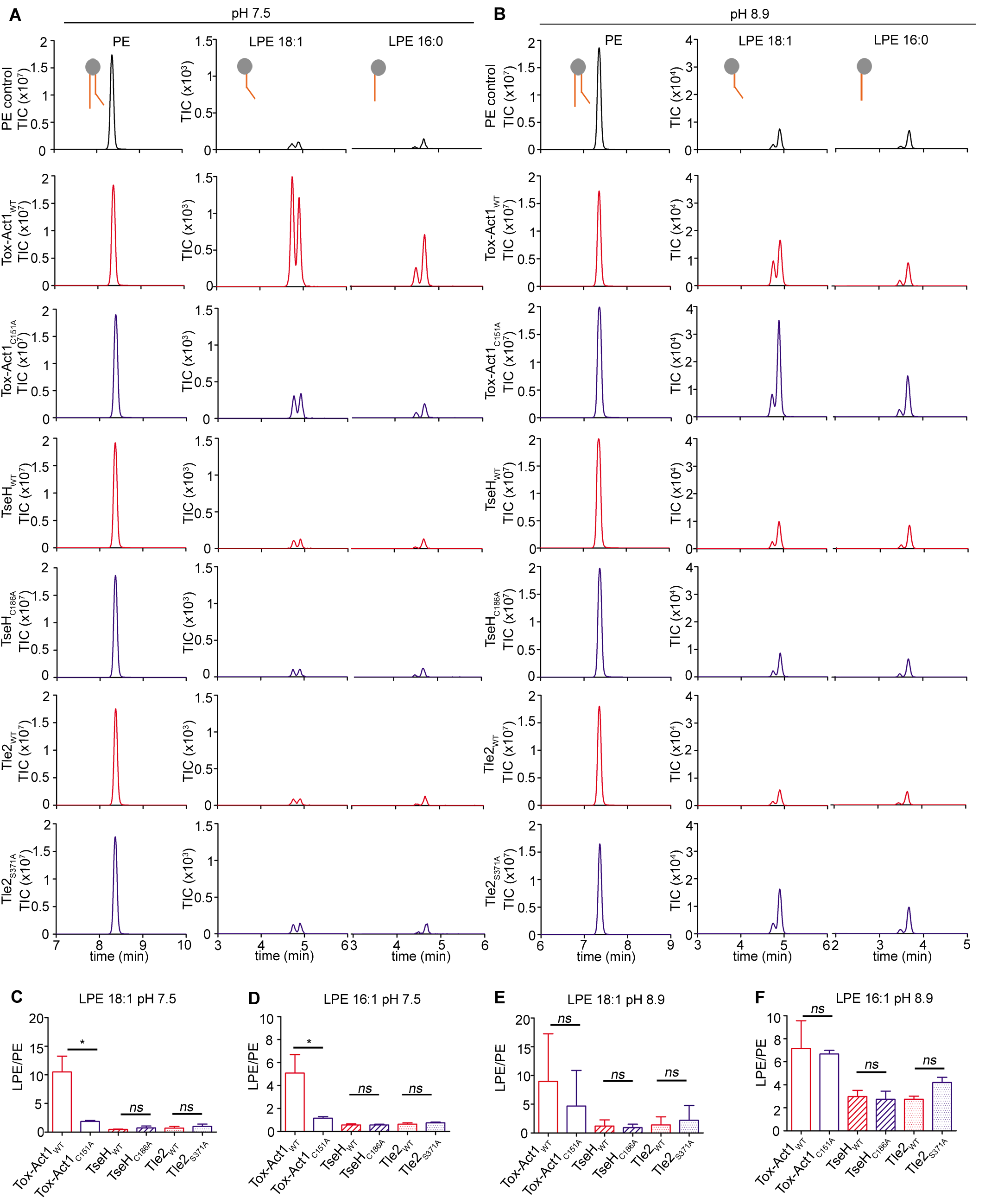

Supplement: S7 Fig — In vitro enzymatic assay with recombinant Tox-Act1, TseH, Tle2 (red) and their respective catalytic mutants Tox-Act1C151A, TseHC186A and Tle2S371A (blue) incubated with 16:0–18:1 PE at pH 7.5 (a) or pH 8.9 (b). The amount of lysophospholipids produced was analyzed by HPLC-MS/MS. (c–f) Quantification of the peak area of lysophospholipids was normalized by the intact substrate. Data corresponds to the mean ± SD. *p < 0.05, ns not significant (Student t test). The source data for this figure can be found in https://zenodo.org/records/18590644/files/10ksgt6ss-10.zip?download=1. (TIF) [file pbio.3003680.s007.tif]

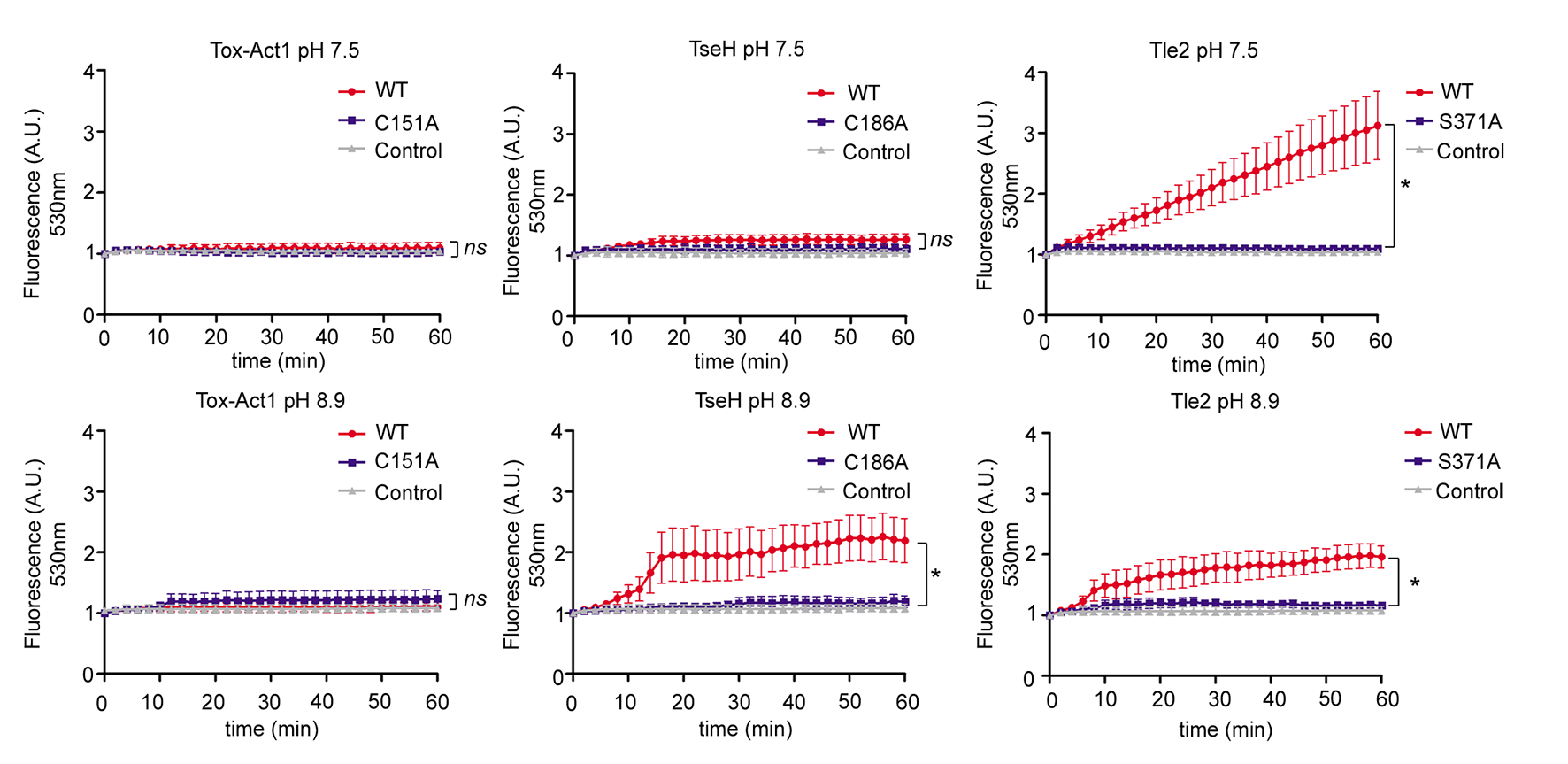

Supplement: S8 Fig — Liposomes containing the fluorescent substrate PED-A1 were incubated with recombinant Tox-Act1, TseH, Tle2 (red) or their catalytic mutants Tox-Act1C151A, TseHC186A and Tle2S371A (blue). Fluorescence is represented in arbitrary units (A.U). Data is the mean ± SD of at least four independent experiments. *p < 0.05 (Student t test). The source data for this figure can be found in https://zenodo.org/records/18590644/files/10ksgt6ss-10.zip?download=1. (TIF) [file pbio.3003680.s008.tif]

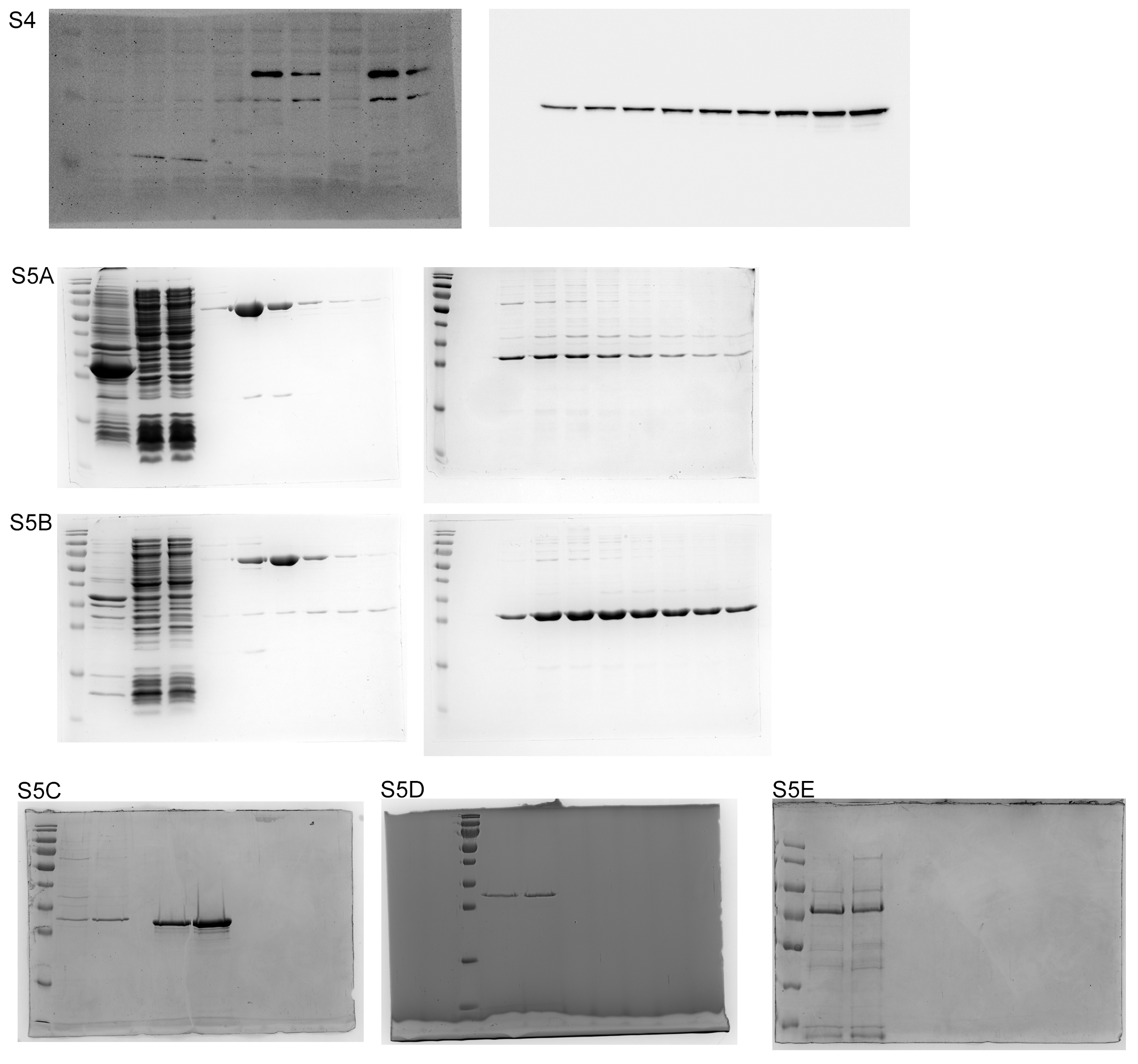

Supplement: S1 Raw Images — (TIF) [file pbio.3003680.s019.tif]
