# Supplementary material for: Systematic identification of Salmonella T6SS effectors uncovers diverse new families and lipid-targeting activities
Source: PLoS Biol. 2026 Mar 17;24(3):e3003680. doi: 10.1371/journal.pbio.3003680 (PMC12994826; doi:10.1371/journal.pbio.3003680)

Phylogenetic trees

- **Blue**: sequences originating from SecReT6; each label shows the classification reported in that dataset.
- **Red**: sequences derived from the 10KSG project; each point label shows the community assignment generated by the Louvain algorithm.

TssA

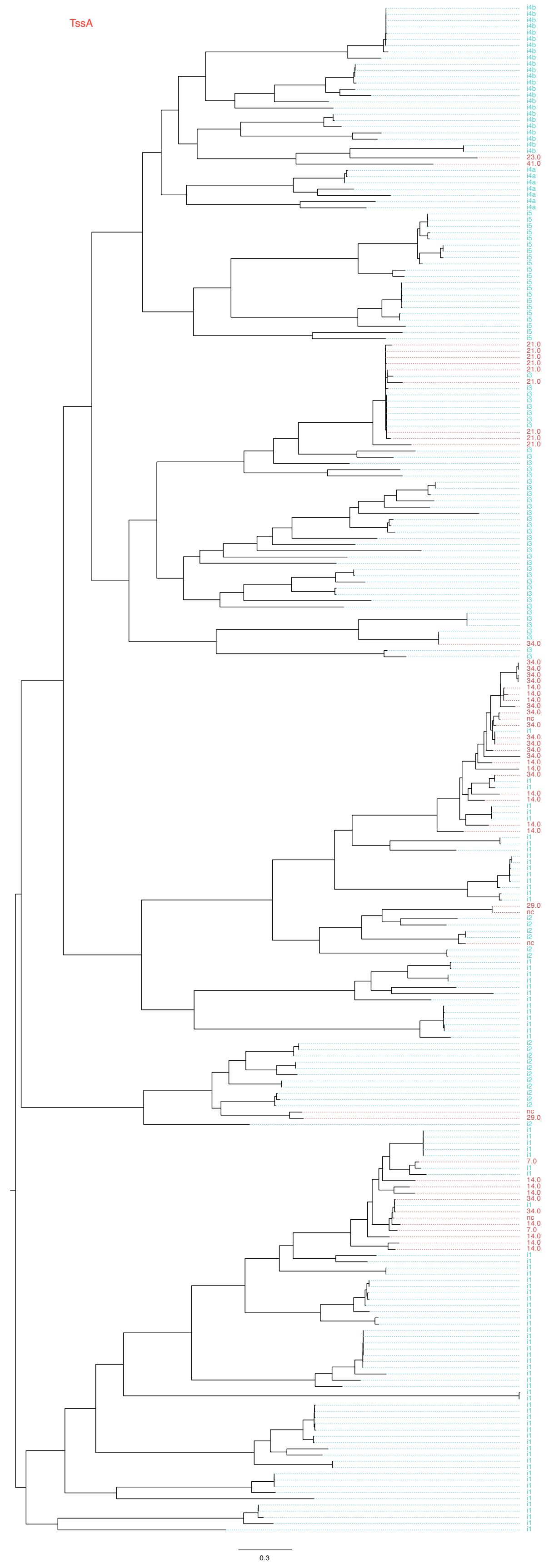

TssB

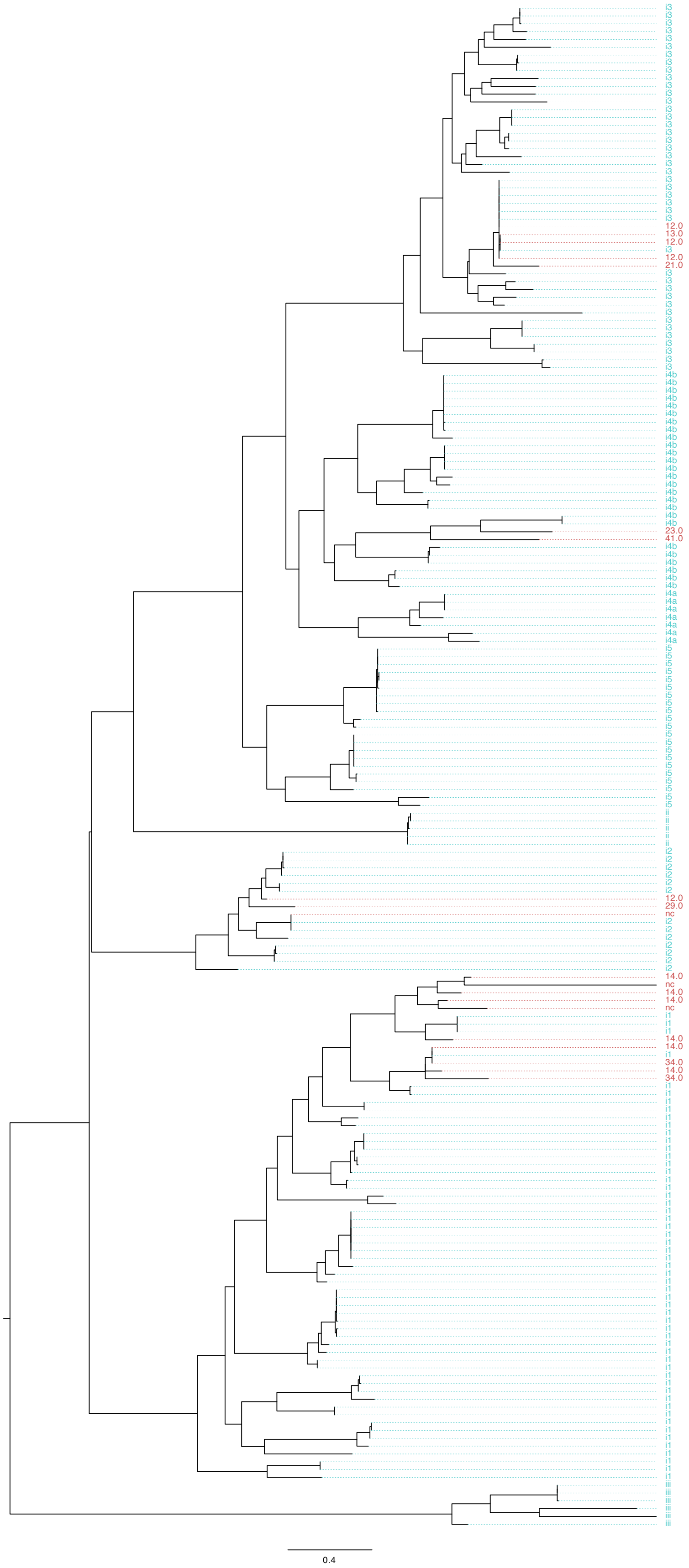

TssC

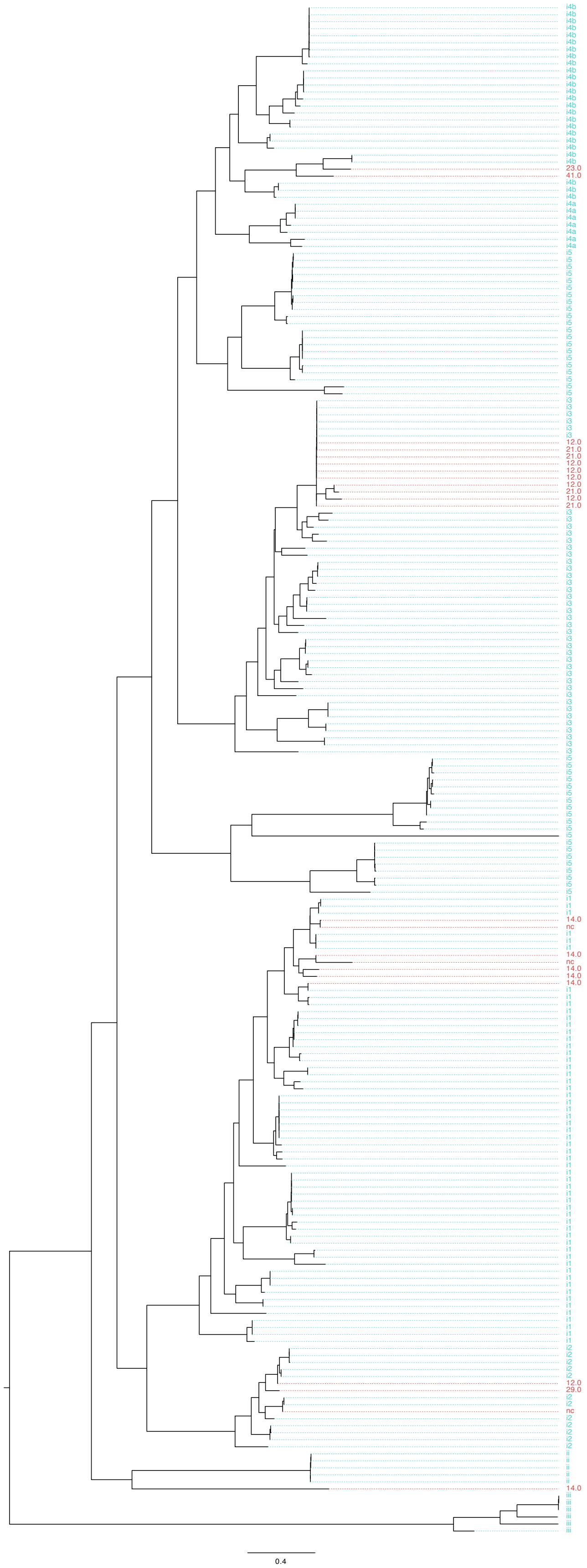

TssE

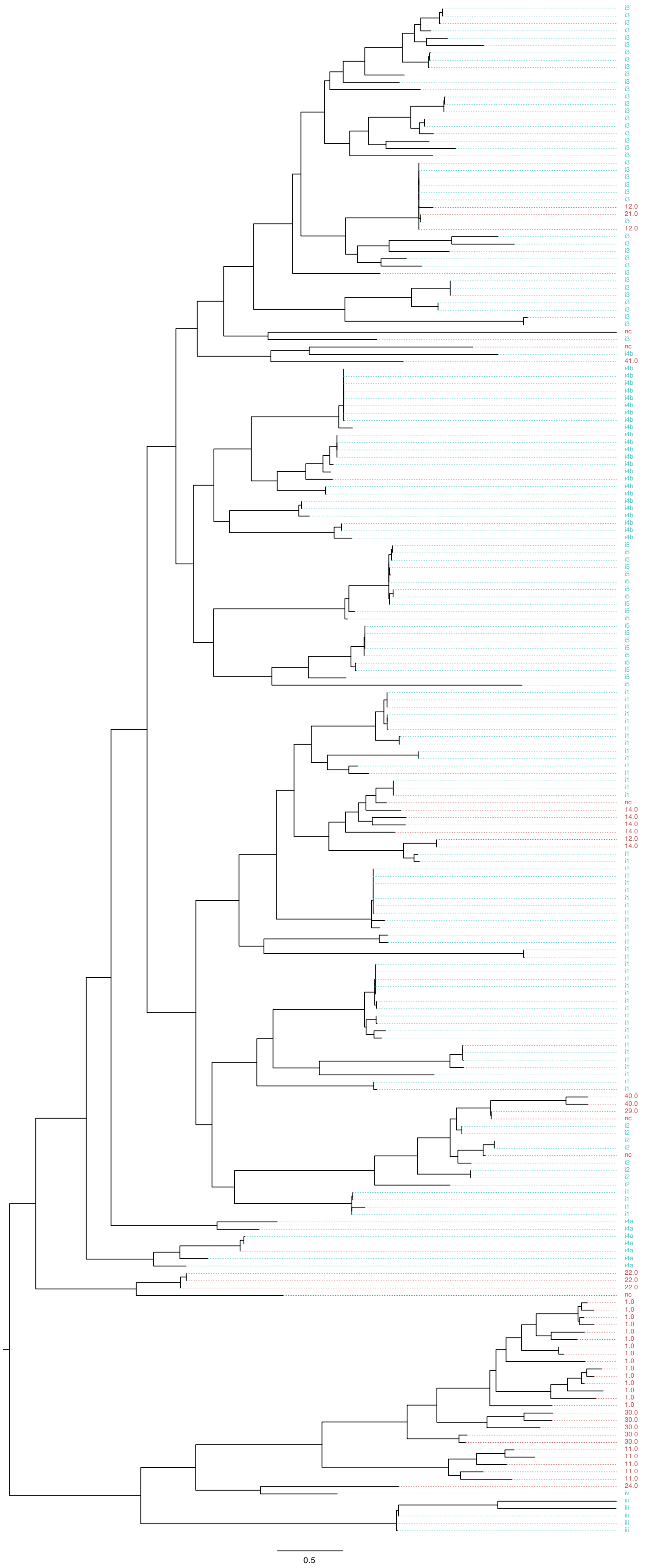

TssF

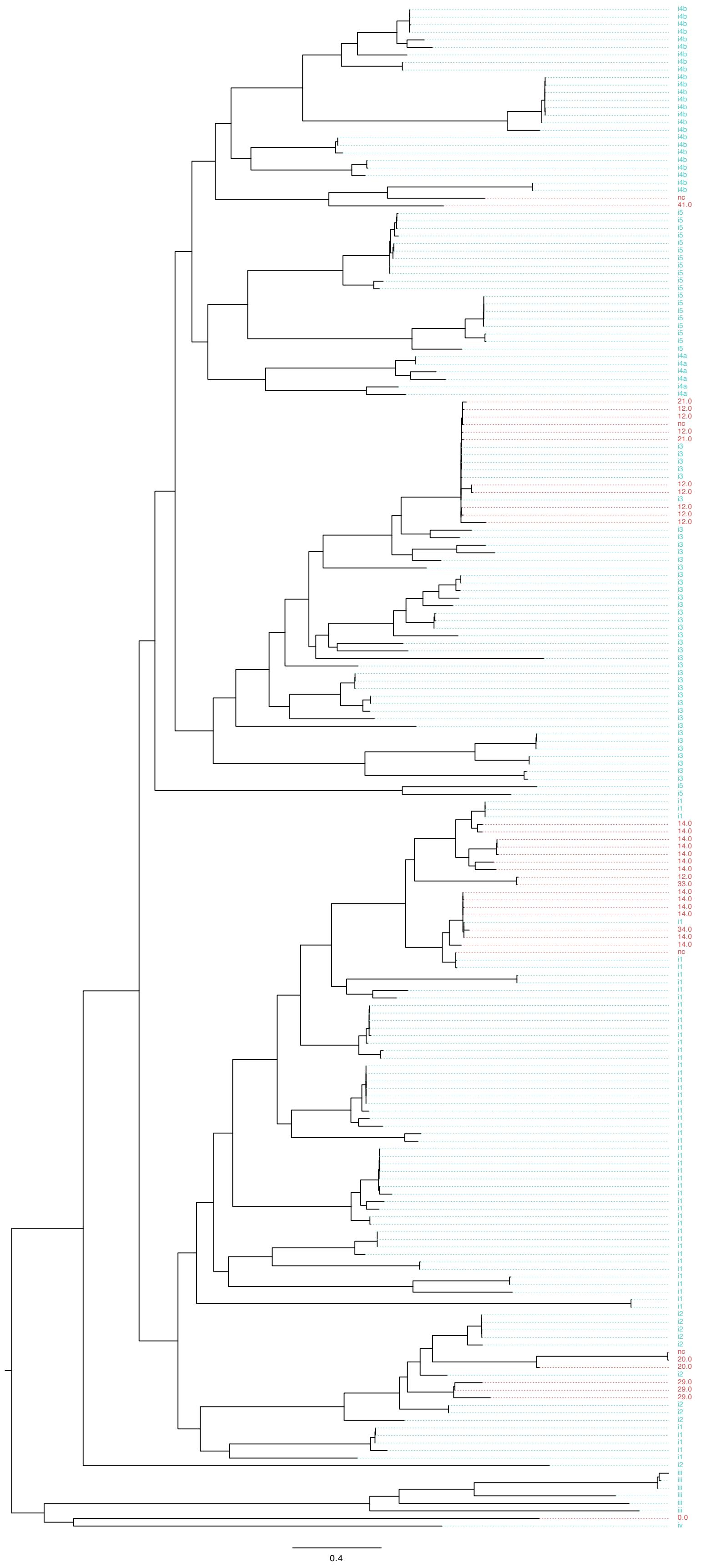

TssG

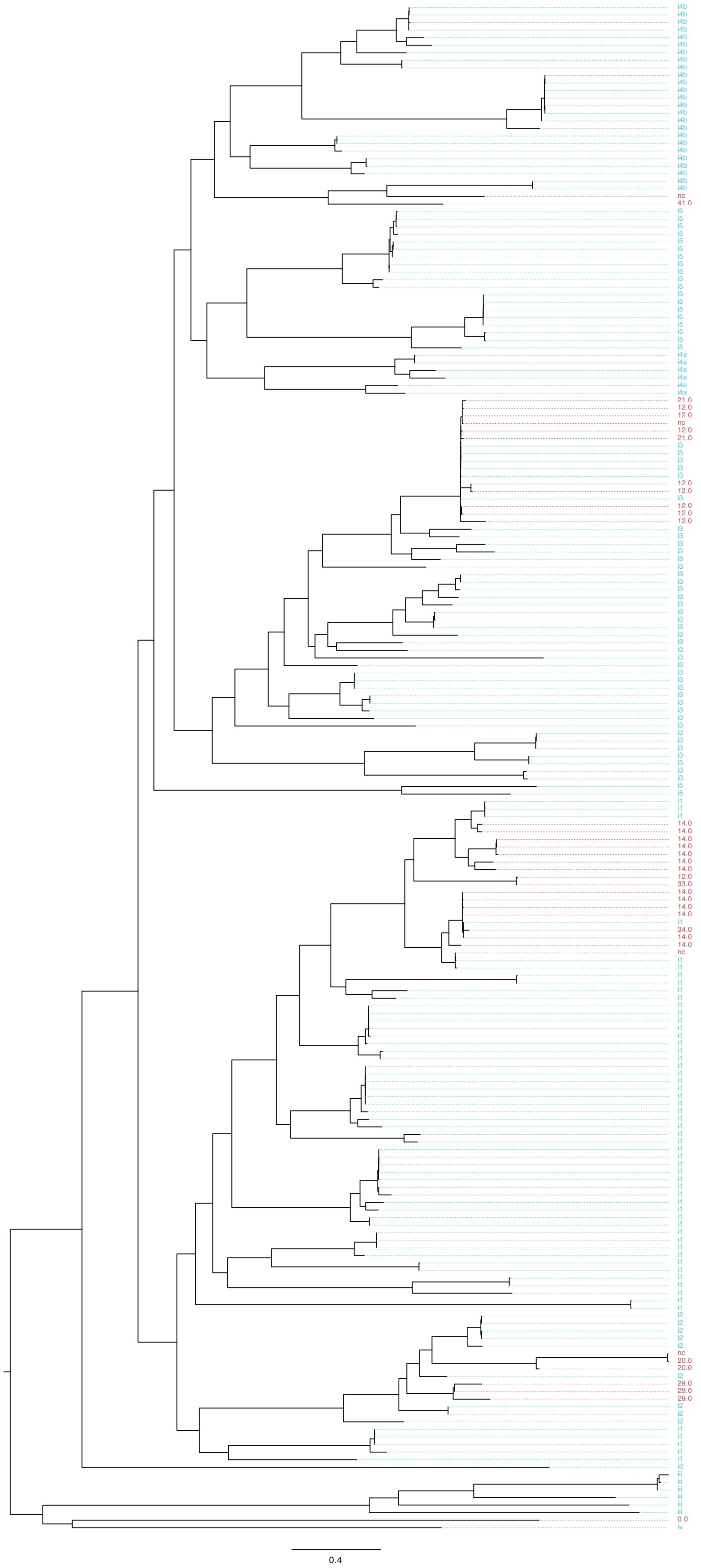

TssH

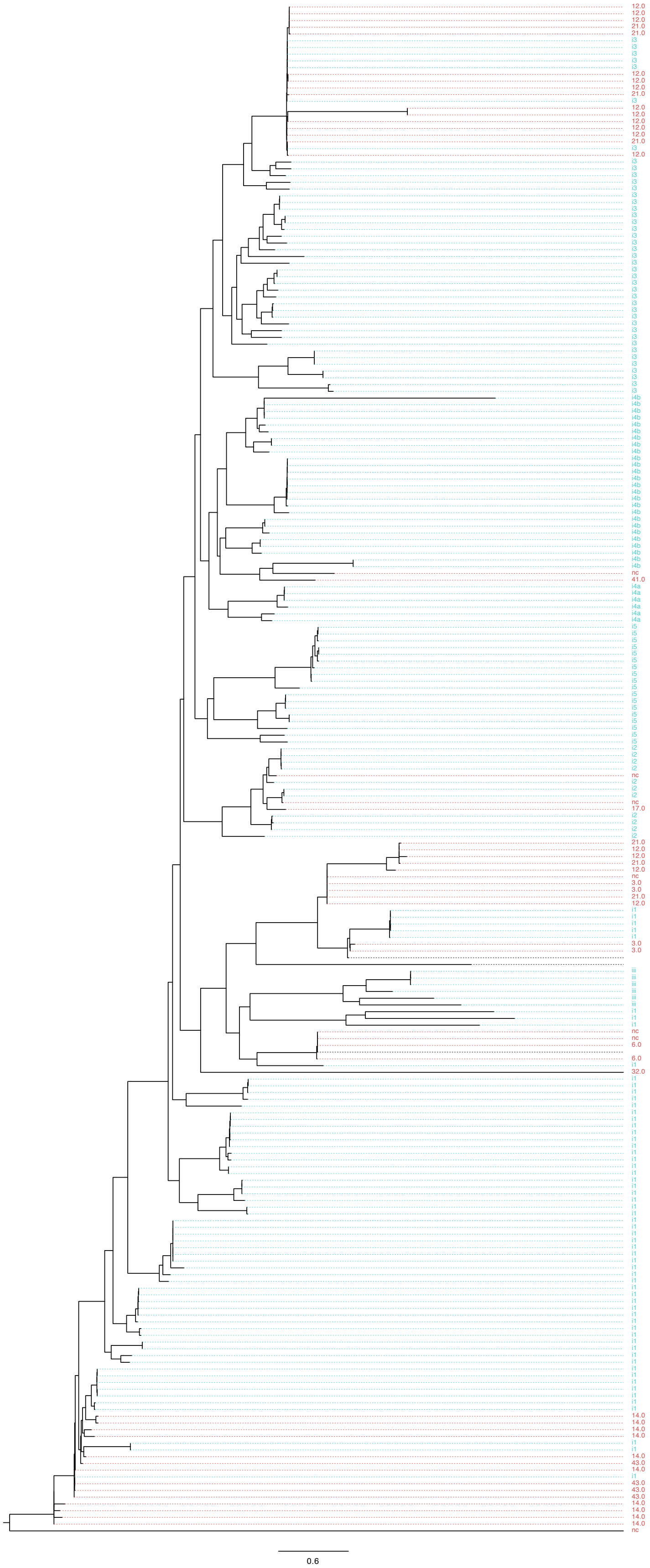

TssI

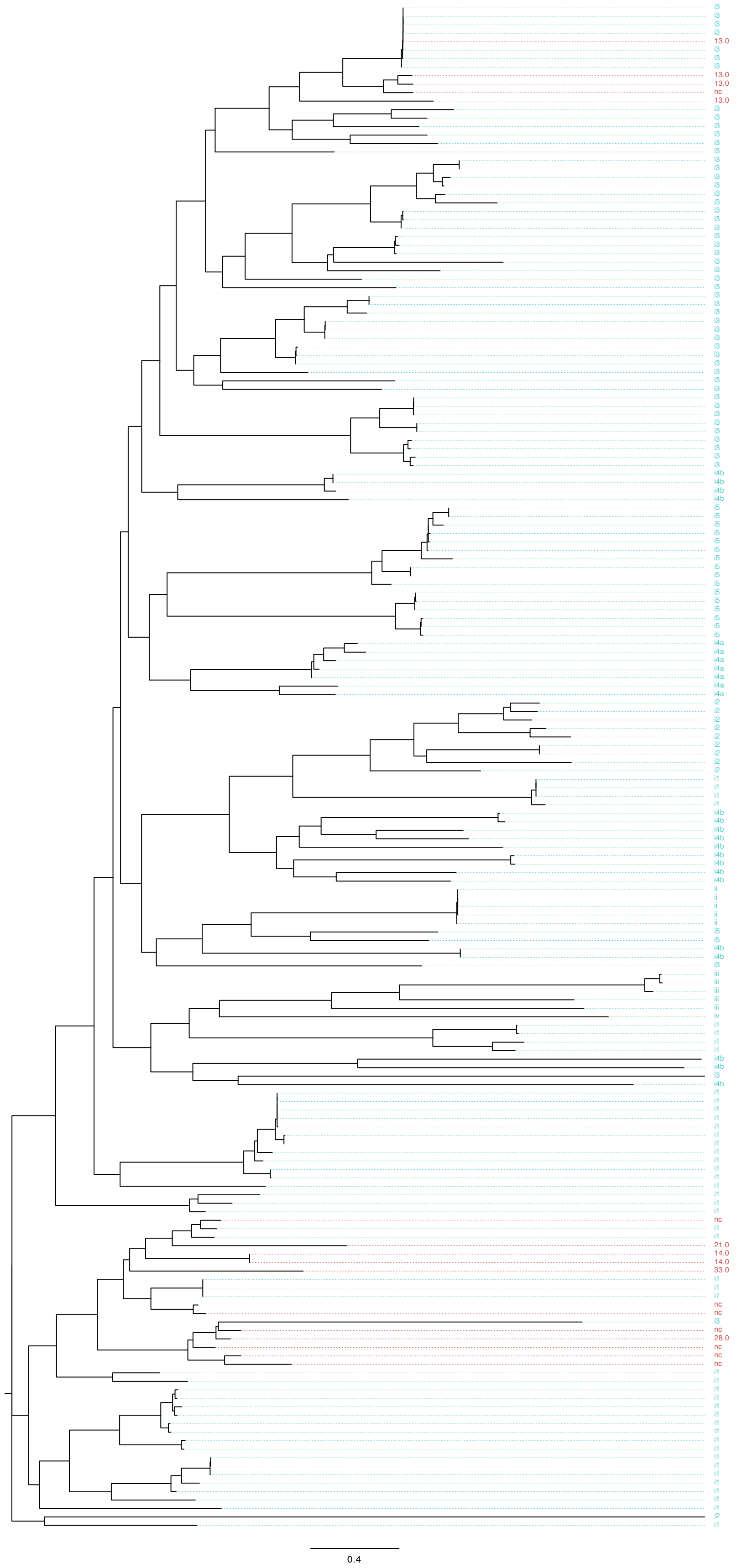

TssJ

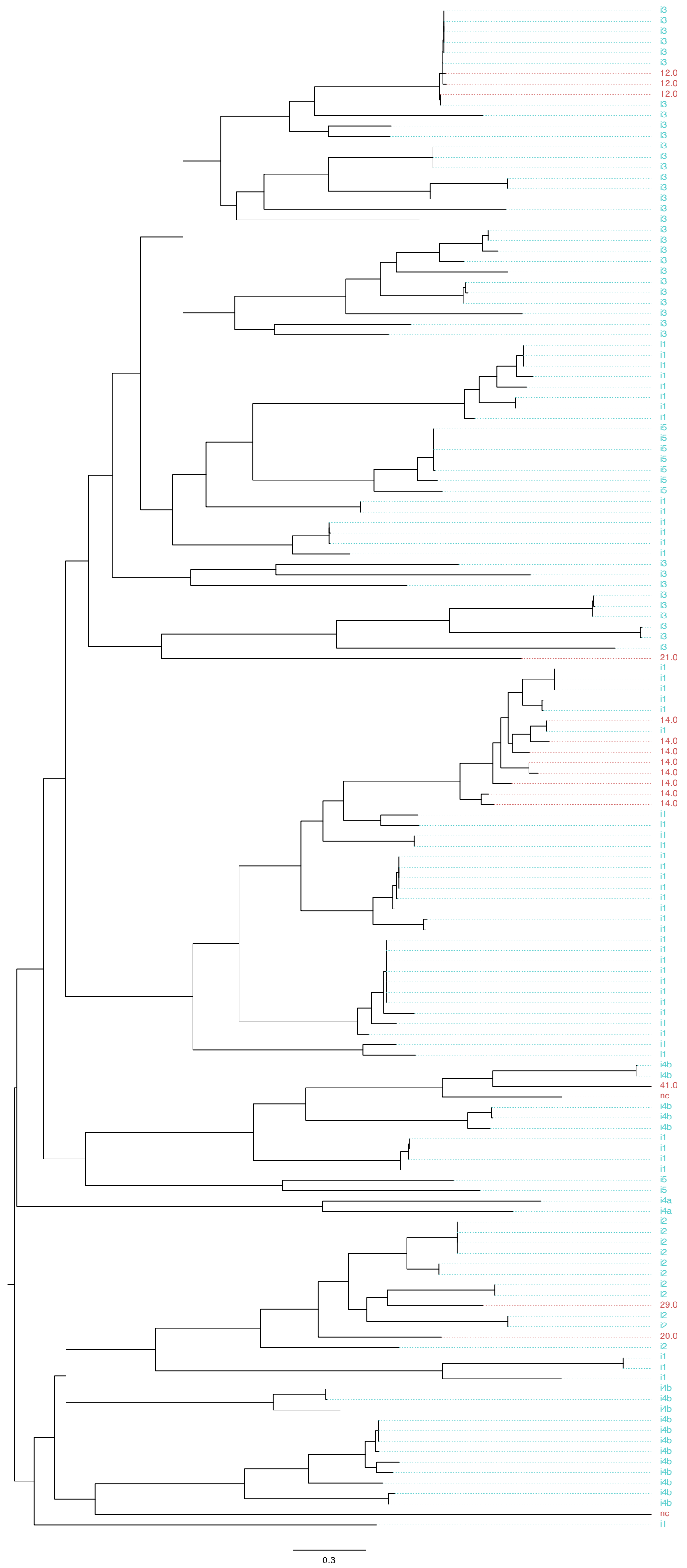

TssK

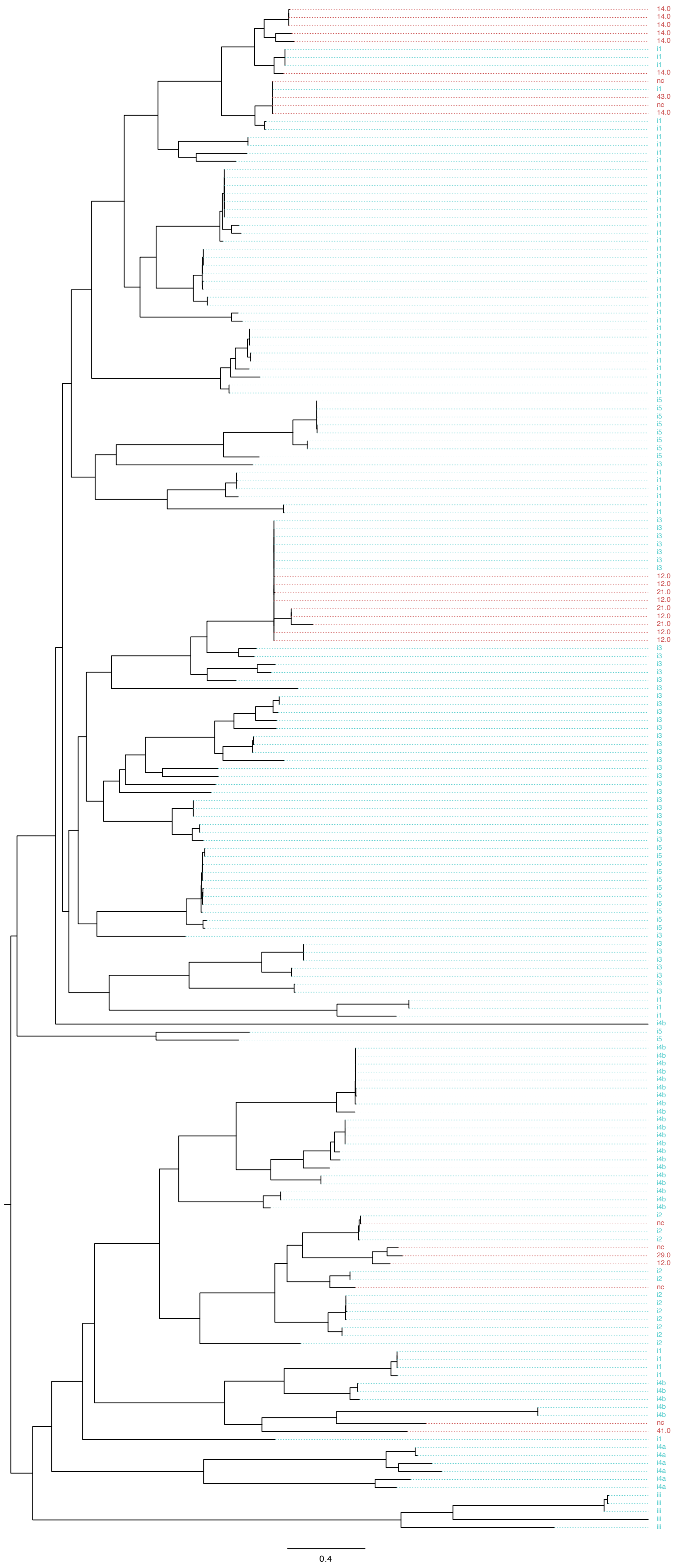

TssL

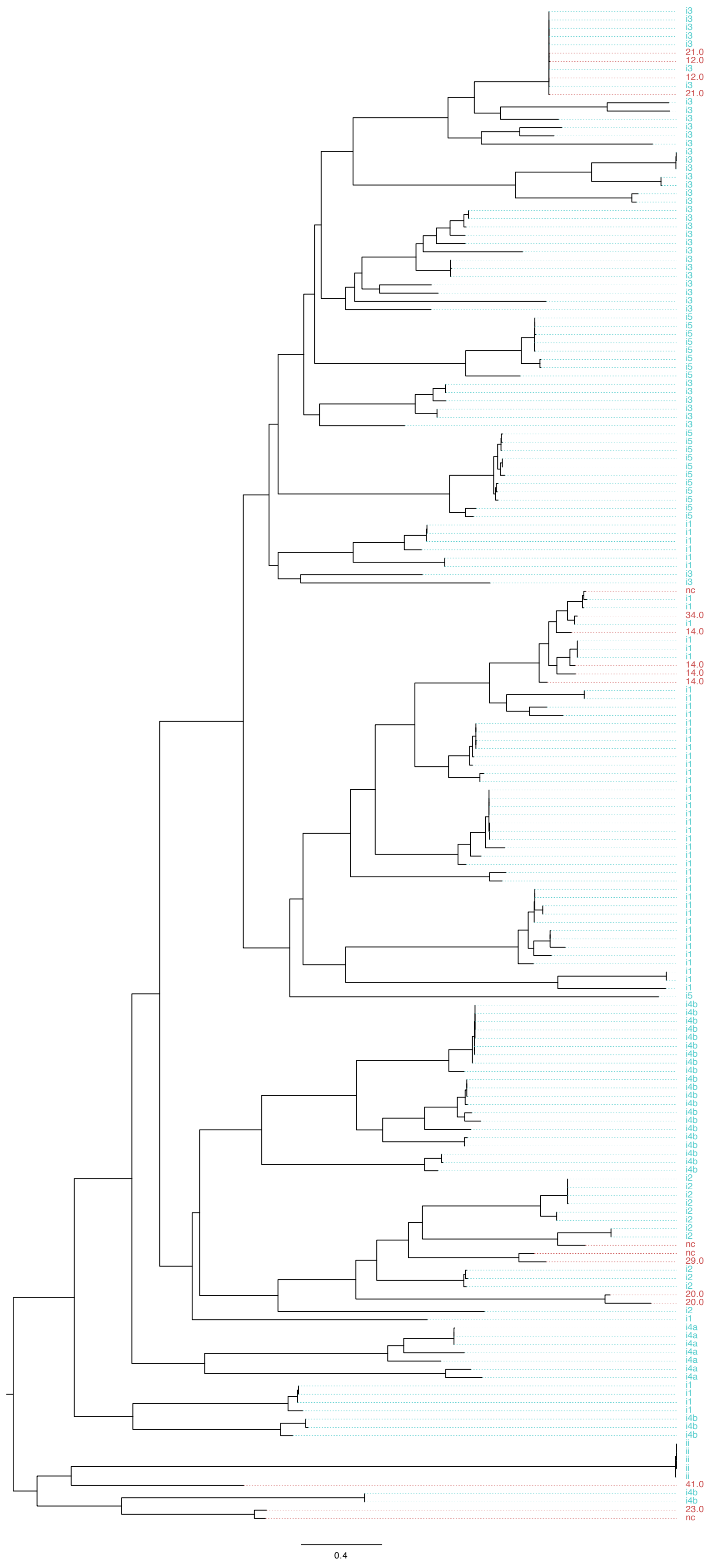

TssM

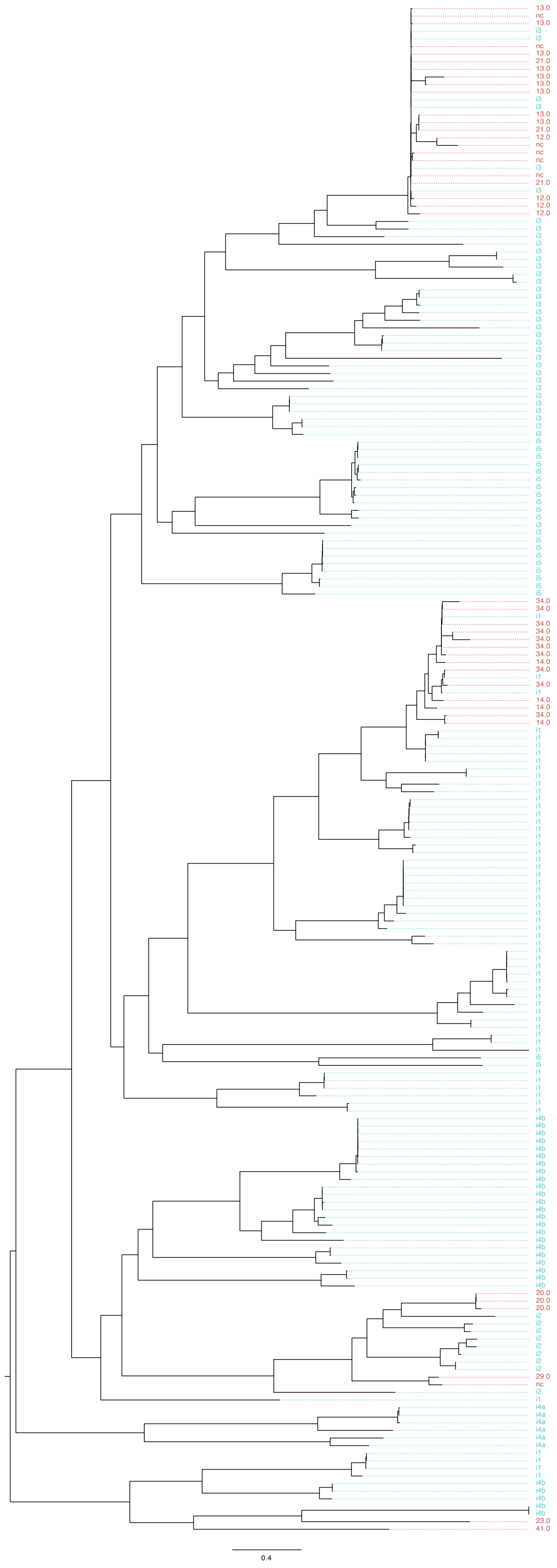

Supplement: S1 Data — Phylogenetic trees are shown for individual T6SS components. Blue labels indicate sequences originating from the SecReT6 dataset [34], with labels showing the classification reported in that dataset. Red labels indicate sequences derived from the 10KSG project, with labels showing the community assignments generated using the Louvain algorithm. (PDF) [file pbio.3003680.s018.pdf]
